# Aerosol-Mediated Non-Viral Lung Gene Therapy: The Potential of Aminoglycoside-Based Cationic Liposomes

**DOI:** 10.3390/pharmaceutics14010025

**Published:** 2021-12-23

**Authors:** Tony Le Gall, Mathieu Berchel, Lee Davies, Angélique Mottais, Rosy Ghanem, Alain Fautrel, Deborah Gill, Steve Hyde, Pierre Lehn, Jean-Marie Lehn, Loïc Lemiègre, Thierry Benvegnu, Paul-Alain Jaffrès, Bruno Pitard, Tristan Montier

**Affiliations:** 1 Univ Brest, INSERM, EFS, UMR 1078, GGB-GTCA, F-29200 Brest, France; angeliquemottais@gmail.com (A.M.); rosy.ghanem@univ-brest.fr (R.G.); pierre.lehn@univ-brest.fr (P.L.); 2 Univ Brest, CNRS, CEMCA UMR 6521, 6 Avenue Victor Le Gorgeu, 29238 Brest, France; mathieu.berchel@univ-brest.fr (M.B.); paul-alain.jaffres@univ-brest.fr (P.-A.J.); 3 Gene Medicine Group, Radcliffe Department of Medicine (Clinical Laboratory Sciences), John Radcliffe Hospital, University of Oxford, Oxford OX3 9DU, UK; lee.davies@ndcls.ox.ac.uk (L.D.); deborah.gill@ndcls.ox.ac.uk (D.G.); steve.hyde@ndcls.ox.ac.uk (S.H.); 4 Univ Rennes, INSERM, CNRS, UMS Biosit, France-BioImaging, Core Facility H2P2, F-35000 Rennes, France; alain.fautrel@univ-rennes1.fr; 5 Laboratoire de Chimie des Interactions Moléculaires, Collège de France, Place Marcelin Berthelot, 75005 Paris, France; lehn@unistra.fr; 6 Univ Rennes, Ecole Nationale Supérieure de Chimie de Rennes, CNRS, ISCR-UMR 6226, 11 allée de Beaulieu, CS 50837, CEDEX 7, 35708 Rennes, France; loic.lemiegre@ensc-rennes.fr (L.L.); thierry.benvegnu@ensc-rennes.fr (T.B.); 7 Univ Nantes, CNRS ERL6001, INSERM 1232, CRCINA, F-44000 Nantes, France; bruno.pitard@univ-nantes.fr; 8 CHRU de Brest, Service de Génétique Médicale et de Biologie de la Reproduction, Centre de Référence des Maladies Rares “Maladies Neuromusculaires”, F-29200 Brest, France

**Keywords:** aminoglycoside-based cationic lipids, cystic fibrosis, lipidic formulations, lung gene therapy, nebulization, structure-activity

## Abstract

Aerosol lung gene therapy using non-viral delivery systems represents a credible therapeutic strategy for chronic respiratory diseases, such as cystic fibrosis (CF). Progress in CF clinical setting using the lipidic formulation GL67A has demonstrated the relevance of such a strategy while emphasizing the need for more potent gene transfer agents. In recent years, many novel non-viral gene delivery vehicles were proposed as potential alternatives to GL67 cationic lipid. However, they were usually evaluated using procedures difficult or even impossible to implement in clinical practice. In this study, a clinically-relevant administration protocol via aerosol in murine lungs was used to conduct a comparative study with GL67A. Diverse lipidic compounds were used to prepare a series of formulations inspired by the composition of GL67A. While some of these formulations were ineffective at transfecting murine lungs, others demonstrated modest-to-very-efficient activities and a series of structure-activity relationships were unveiled. Lipidic aminoglycoside derivative-based formulations were found to be at least as efficient as GL67A following aerosol delivery of a luciferase-encoding plasmid DNA. A single aerosol treatment with one such formulation was found to mediate long-term lung transgene expression, exceeding half the animal’s lifetime. This study clearly supports the potential of aminoglycoside-based cationic lipids as potent GL67-alternative scaffolds for further enhanced aerosol non-viral lung gene therapy for diseases such as CF.

## 1. Introduction

Nebulization to the lung airways is an attractive, clinically-relevant, administration method, especially because high loads of active drug can be directly delivered to the respiratory epithelium. It is particularly suitable to treat various respiratory diseases, notably cystic fibrosis (CF; OMIM 219700; one of the most prevalent genetic diseases in Caucasian populations). The latter is a life-threatening autosomal recessive disorder that has a prognosis closely associated with the respiratory failure it causes [1]. Along with tremendous recent steps towards CF precision medicine with targeted pharmaceutical treatments [2], lung gene therapy using aerosolized synthetic or viral carriers remains a credible strategy for that disease [3]; specifically, it would have the important advantage of being applicable to all CF patients, regardless of their mutation profile. Encouraging progress has been achieved supporting further developments in this approach [4,5]. However, the airway delivery route also faces specific obstacles, stimulating research focusing on both the inhalation method [6] and the therapeutic treatment to be delivered [7,8,9].

**Figure 1:**
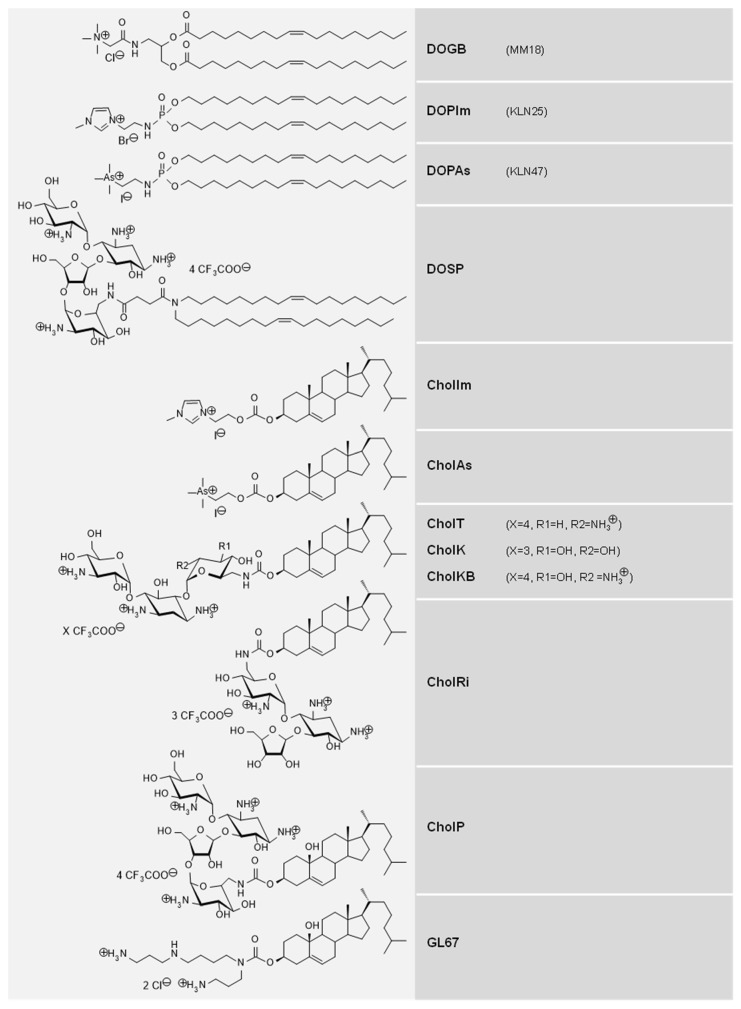
Chemical structure of the cationic lipids considered in this study. DOGB, dioleyl glycine betaine; DOPIm, dioleyl phosphoramide imidazolium; DOPAs, dioleyl phosphoramide arsonium; DOSP, dioleyl-succinyl paromomycin; CholIm, cholesterol imidazolium; CholAs, cholesterol arsonium; CholT, cholesterol tobramycin; CholK, cholesterol kanamycin A; CholKB, cholesterol kanamycin B; CholRi, cholesterol ribostamycin; CholP, cholesterol paromomycin; GL67, Genzyme (Cambridge, MA, USA) lipid 67 [10,28,29,30,32,33].

**Figure 2:**
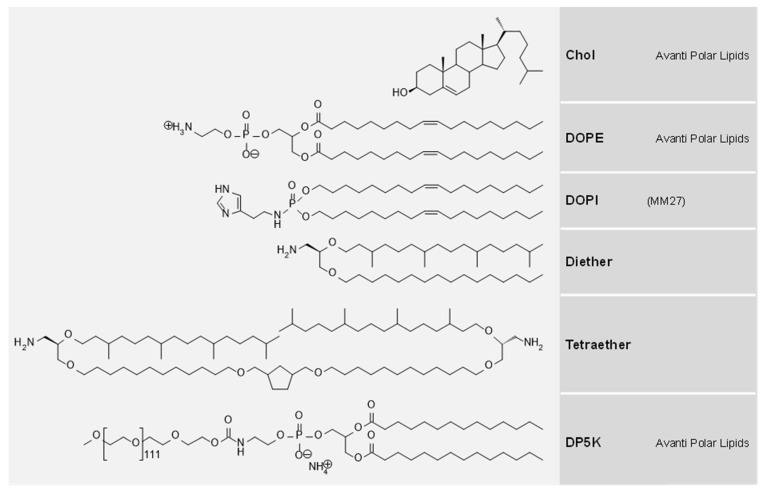
Chemical structure of other compounds used as colipids in this study. Chol, cholesterol; DOPE, dioleyl phosphatidylethanolamine; DOPI, dioleyl phosphoramide imidazole; DP5K, 1,2-dimyristoyl-sn-glycero-3-phosphoethanolamine conjugated polyethylene glycol 5000 [17,23,34,35].

The liposome-based formulation GL67A is still considered as the lead synthetic vector for non-viral CF gene therapy. It consists of a combination of (1) an amphiphile incorporating a cholesterol anchor linked to a spermine headgroup (the so-called GL67 cationic lipid), (2) a zwitterionic colipid (the dioleyl phosphatidylethanolamine, classically shortened to DOPE), and (3) a steric stabilizer (the 1,2-dimyristoyl-sn-glycero-3-phosphoethanolamine conjugated polyethylene glycol 5000, hereafter abbreviated as DP5K) (Figure 1 and Figure 2) [10,11]. This formulation has been evaluated extensively by the UK Cystic Fibrosis Gene Therapy Consortium in pre-clinical [10,12,13,14] and clinical [7,15,16] settings. The most recent non-viral CF gene therapy clinical trial consisted of a randomized, double-blind, placebo-controlled, phase 2b trial of repeated nebulization of GL67A liposomes [4]. It demonstrated for the first time that patients carrying a broad range of mutations can be repeatedly treated with a non-viral gene therapy without showing any adverse effects; significant beneficial effects on lung function (as shown by stabilization of the forced expiratory volume in one second, FEV_1_) were observed in patients treated with GL67A compared with patients who received a placebo [4]. However, this trial also stressed that further work needs to be done in order to obtain more substantial clinical benefits. This implies the need to identify more efficient alternatives to GL67A.

In recent years, a plethora of synthetic gene carriers for lung-directed gene therapy has been investigated. For our part, we have reported and evaluated a wide range of original bio-inspired cationic or neutral lipids, under various in vitro [17,18,19] and in vivo [20,21,22,23,24] conditions. In particular, we have demonstrated efficient lung transfection in mice using one of our vectors in a multiple administration procedure [25]. It is noteworthy that in many of these in vivo studies, systemic injection via the tail vein of mice was used as a convenient administration procedure to estimate the ability of these vectors to deliver genes to the lungs. However, despite this route allowing transfection of pulmonary epithelial cells relevant for CF (notably type I and type II pneumocytes [22]), its use in humans is not credible and ethically acceptable, since multiple organs can be exposed to a potentially toxic treatment. Other delivery routes have been investigated for the development of non-viral airway gene transfer but these also showed limitations [26]. Ultimately, due to the use of different experimental delivery protocols or to non-standardized comparator references, it remains difficult to assess whether any of the many gene transfer agents currently described actually offer an advantage, especially compared with GL67A, for aerosol delivery.

In this study, eleven cationic lipids (Figure 1) and five colipids (Figure 2) with structural diversity (compared with both GL67 and DOPE) were used to prepare a series of lipidic formulations. Their gene delivery properties were assessed using a clinically relevant administration protocol via nebulization to murine lungs, with GL67A evaluated in parallel for direct comparison. Since the experimental conditions were the same throughout the study, it was possible to accurately compare the gene delivery potential of the respective formulations. This led to the identification of aminoglycoside derivative-based formulations capable to challenge GL67A under experimental conditions relevant for CF.

## 2. Materials and Methods

### 2.1. Chemicals and Reagents

Previously reported cationic lipids (Figure 1) were synthetized according to published procedures with some optimizations; these included DOGB (also known as MM18) [23], DOPIm (KLN25) [27], DOPAs (KLN47) [20], DOSP [28,29], CholK [30,31,32], CholKB [30,31,32], CholP [29], CholRi [29], and CholT [33]. Original cationic lipids—namely CholIm and CholAs—were obtained following synthetic routes detailed in supporting information. Compounds used as colipids (Figure 2) were purchased from Avanti Polar Lipids (Cholesterol, DOPE, and DP5K; Alabaster, AL, USA) or they were synthetized as previously described (DOPI (MM27) [17], Tetraether [23,34], and Diether [35]). Clinical-grade batches of GL67A (07D26701-01A, OctoPlus NV) were kindly provided by Seng H. Cheng.

### 2.2. Plasmid DNA

Two different plasmid DNA (pDNA) were used: either the CpG-free firefly luciferase-encoding pGM144 [36] or the human CFTR (hCFTR)-encoding pCIK-CFTR (originally described in [37]). The former pDNA (pGM144, 3759 bp) was designed to limit inflammation and to mediate high and sustained transgene expression in vivo [36]. It contains a synthetic codon-optimized firefly luciferase transgene, driven by the human Elongation Factor 1α promoter with a human cytomegalovirus (CMV) enhancer, a CpG-free intron, a BGH Poly(A) sequence, and a kanamycin resistance gene. It was amplified and purified by Aldevron (Madison, WI, USA), in endotoxin-free water with 8 mM of NaCl (production lots 55,883 and 15,804). Unlike pGM144, pCIK-CFTR (8520 bp) corresponds to a CpG-containing pDNA, in which an hCFTR transgene is driven by the human CMV immediate-early promoter. It also contains a hybrid intron and a Kozak sequence downstream of the promoter, a SV40 Poly(A) sequence, and an ampicillin resistance gene. It was amplified and purified using NucleoBond PC 10,000 EF (Macherey Nagel, Hoerdt, France). In every instance, pDNA was used only if it was predominantly supercoiled, containing less than 5 EU endotoxin/mg, and highly concentrated (≥5 mg/mL) with high purity (OD_260/280_ ≥ 1.8 and ≤2.0).

### 2.3. Animal Experimentation: Ethical Approval and Experimental Animals 

All the procedures were carried out with personal licenses under protocols approved by the Laboratory Animal Care Guidelines and the Institutional Animal Care and Research Advisory Committee at both the University of Oxford (UK) and the University of Brest (France). Furthermore, the experiments were in accordance with the ARRIVE guidelines [38]. Female BALB/c mice were purchased from the Biomedical Services Unit of the University of Oxford (Oxford, UK) or from Janvier Breeding Center (Le Genest-Saint-Isle, France). Male or female *Cftr*^−/−^ mice were obtained from the CDTA-CNRS (Orléans, France). They derived from *Cftr*^tm1Unc^ knockout mice homozygous for the S489X-CFTR mutation [39] back-crossed for three generations into C57BL/6. All the animals were housed in accordance with the recommendations of regional veterinary services. They enjoyed free access to drinking water and were fed ad libitum on a standard diet, under a 12/12 h light/dark cycle. Specifically, the *Cftr*^−/−^ mice were kept in a ventilated cabinet and their drinking water was supplemented with an osmotic laxative (Movicol^®^; Norgine, Rueil-Malmaison, France) containing polyethylene glycol and electrolytes (to minimize bowel obstruction and optimize viability). In any case, acclimatization lasted at least 1 week and the animals were 6 to 9 weeks old at the time of the procedure.

### 2.4. Preparation of Liposomes

Each formulation was prepared as a liposomal solution using the lipid film hydration method, as reported previously [20,23,28]. Briefly, for a given lipidic formulation, a concentrated stock solution in chloroform (Sigma Aldrich, Saint-Quentin-Fallavier, France) was prepared for each cationic lipid and colipid(s) to be used. The required volume of each of the latter was then introduced in a glass vial and the solvent was evaporated under vacuum to obtain a thin lipid film. A volume of water was then added over the latter. After hydration for at least 12 h at 4 °C, the mixture was subjected to repeated cycles of homogenization, i.e., it was vortexed (for 1 min) then sonicated (for 10 min) in an ultrasonic water bath; this was repeated at least five times. Unless otherwise stated, supplementation with DP5K was performed thereafter by adding the required volume of this compound previously dissolved in water. The resulting liposomal solution was stored at 4 °C until subsequent use. The composition of the various liposomes prepared is detailed in the Results section.

### 2.5. Preliminary Physicochemical Characterizations of Liposomes and Lipoplexes

For the liposomes, the possibility to combine within a single formulation given cationic lipids and colipids at various molar ratios was first investigated. For the lipoplexes, preliminary testing was performed by gently mixing 250 µg pDNA in 50 µL with 50 µL of a given liposome, in order to obtain lipoplexes featuring characteristics identical to those needed for conducting in vivo aerosol evaluations. The charge ratio (CR) is the ratio of positive charges provided by the cationic lipid to the negative charges provided by the pDNA; complexes were prepared for CR between 1 and 4. The PEGylation ratio (PR) corresponds to the DP5K to pDNA mass ratio; this parameter varied between 0.5 and 3.0. For both the liposomes and the lipoplexes, homogeneity was first examined by visual inspection. For the homogeneous mixtures, the size and zeta potential were measured at room temperature (RT) using a Zetasizer instrument (Malvern, Palaiseau, France).

### 2.6. Preparation of pDNA Lipoplexes for Aerosol Delivery

For performing an aerosol experiment in mice, the lipoplexes were prepared extemporaneously in 10 aliquots, by gently mixing 500 µL (2.5 mg pDNA) with 500 µL of lipidic formulation. After 15 min at RT, the aliquots were pooled to obtain 10 mL of solution containing 25 mg of complexed pDNA. 

### 2.7. Aerosol Delivery

**Figure 3:**
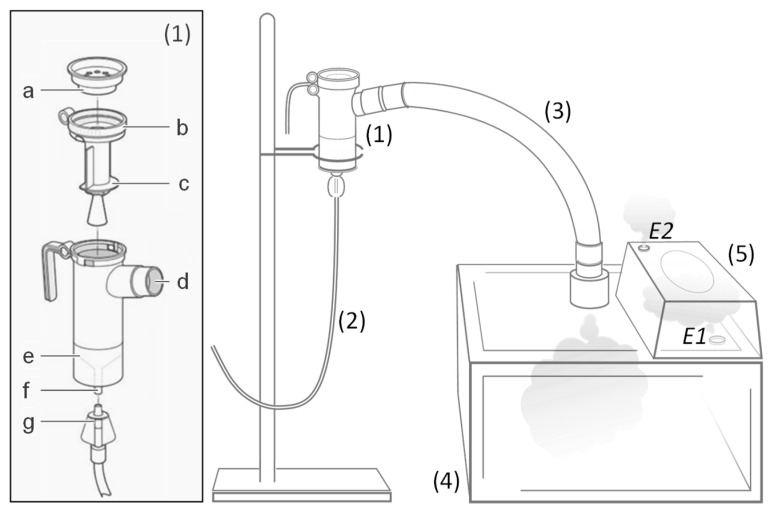
Aerosol experimental set-up. The illustration shows the clinically-validated PARI LC PLUS nebulizer (1) (comprising: a, inspiratory valve cap; b, insert; c, semi-circle; d, outlet; e, reservoir; f, air intake; g, wing tip tubing) connected on one side to a flexible hose (supplying two bars pressurized air) (2) and on the other side to a 55 cm tube (3) conducting the aerosol generated to a Perspex box (4) where mice were housed. Via an exit, E1, the aerosol reached a second compartment (5) fitted over the cover of the exposure box. This second compartment made it possible to expose other materials and to collect a sample of the aerosolized formulation. The aerosol finally left the system via an exit, E2. See Figure S1 for more details about the set-up. A video showing an aerosol experiment in mice is available from (“*Aerosol in mice.mp4*”). https://susy.mdpi.com/user/submission/video/916a160f3cc7e836c26793ade8089553 (accessed on 29 November 2021).

The aerosol delivery was performed using an unrestrained whole-body exposure protocol with the PARI LC PLUS nebulizer (PARI, Starnberg, Germany), which has been tested and proven in many clinical trials (Figure 3; see also Figure S1 for more details about the experimental set-up) [8,9]. For each aerosol experiment, 15 to 20 mice were introduced into the exposure box. The pDNA lipoplex mixture was placed in the nebulizer reservoir (50 µL being kept aside for complementary analyses; see below). An additional compartment fitted on the cover of the main exposure chamber was used to place a cup (allowing to collect a sample of the aerosol) and in some cases cell cultures (for evaluating in vitro transfection in parallel). The aerosol was generated using the nebulizer operating at 40 psi with compressed air as the driving gas. After treatment, the mice were randomly distributed into three to four groups (five animals each) and appropriately maintained until subsequent analyses (see below). When used, the cell cultures were incubated at 37 °C in a humidified atmosphere with 5% CO_2_ for 48 h before evaluation.

### 2.8. Gel Retardation Assays

The aliquots from the lipoplexes collected before and after nebulization (corresponding to lipoplexes that either remained in the nebulizer reservoir or reached the exposure box) were assayed by agarose gel electrophoresis to assess pDNA compaction and integrity. Dextran sulfate was also used to evaluate the pDNA entrapped within complexes following dismantling of the latter.

### 2.9. Analysis of Luciferase Expression in Living Animals

In vivo bioluminescence was done using non-invasive imaging of luciferase activity performed at regular time intervals (from 7 up to 397 days) following aerosol treatment. Before bioluminescence imaging (BLI), the chest of each animal was shaved and an intraperitoneal injection of luciferin (4 mg in 200 µL Hepes Buffer (20 mM); Interchim, Montluçon, France) was performed. Three minutes later, the animals were anesthetized with a 4% air-isofluran blend. Once laid in the acquisition chamber, the animals were kept anesthetized with a 2% air-isofluran blend. Five minutes after luciferin injection, luminescence images were acquired using the NightOWL II LB983 NC320 in vivo imaging system (Berthold Technologies, Bad Wildbad, Germany) and WinLight 32 associated software (Berthold). The luminescence images were superimposed onto still images of each mouse. The signals were quantified in the unit of photons/sec in regions of interest.

### 2.10. Analysis of Luciferase Expression in Lung Homogenates and Cell Lines

Unless otherwise stated, the animals were killed by cervical dislocation 24 h, 7 days, or 14 to 28 days after aerosol treatment. The lungs and trachea were harvested en bloc and stored at −80 °C. After thawing, tissues were crushed in 1 mL of Passive Lysis Buffer (Promega, Charbonnières-les-Bains, France) using a FastPrep FP120 sample disrupter (Thermo Electron, Villebon-sur-Yvette, France) incorporating Lysing Matrix D sample tubes (MP Biomedicals, Illkirch-Graffenstaden, France). After the centrifugation of homogenates, reporter activity in supernatant was measured using the Luciferase Assay System (Promega). The total protein content was determined using the BCA assay kit (Interchim, Montluçon, France). Normalized luciferase activity was expressed in relative light units (RLU) per mg of total protein. As for cells grown in vitro, they were lysed 36 to 48 h after aerosol exposure, luciferase and protein assays being performed as previously described [18].

### 2.11. Histology

The mice were sacrificed and the lungs were gently perfused via the trachea with 4% paraformaldehyde (PFA; Sigma Aldrich, Saint-Quentin-Fallavier, France). The lungs, trachea, and heart were carefully removed en bloc and introduced in 10 volumes of 4% PFA. Paraffin-embedded tissue was cut (4 µm thick), mounted on positively-charged slides and dried at 58 °C for 60 min. (i) For anti-luciferase immunofluorescence staining, the Discovery XT Automated IHC stainer was used with the RUO discovery Rhodamine detection kit (Roche Diagnostics, Rotkreuz ZG, Switzerland). Each step of the procedure was optimized. The slides were rinsed between steps with a Ventana Tris-based reaction buffer. Following deparaffination with Ventana EZ prep solution at 75 °C for 8 min, antigen detection was performed using Ventana proprietary, Tris-based buffer solution CC1 antibody, at 95–100 °C for 32 min. After rinsing, the slides were incubated at 37 °C for 32 min with 2.5 µg/mL of anti-luciferase rabbit antibody (Nordic, Susteren, The Netherlands). Detection signal enhancement was performed using a RUO discovery Rhodamine detection kit (Roche Diagnostics), with secondary antibody OmniMap Anti-Rabbit HRP incubation for 16 min at 37 °C. DAPI staining (1/500 for 3 min at RT) was performed to visualize the nucleus. (ii) For hematoxylin and eosin (H&E) staining, a LEICA ST5020 autostainer was used (Leica Biosystems, Nanterre, France). Deparaffination was performed with three baths of xylene and rehydration with three baths of 100, 90, and 70% ethanol, respectively. Tissues were stained with a solution of Hemalun Gill II (Merck, Molsheim, France) for 3 min, then washed with H_2_O, followed by bluing in a solution of lithium carbonate for 1 min, then washed with H_2_O. Staining was then carried out with eosin Y ethanol solution for 3 min. Finally, the tissues were dehydrated in ethanol and xylene baths and mounted with a coverslip.

### 2.12. Additional Experimental Details

More experimental details (about in vitro cell cultures, in vitro transfection assays, luciferase and cell viability assays, transaminase assays, CFTR western blot, antibacterial assays) are available in Supplementary Materials.

### 2.13. Statistical Analysis

Non-parametric unpaired Mann–Whitney tests were performed using Prism software version 6.00 (GraphPad, San Diego, CA, USA). A difference was considered statistically significant for a calculated *p*-value ≤ 0.05.

## 3. Results

### 3.1. A Rational Testing Plan Was Followed 

This study included lipid compounds featuring chemical diversity according to the headgroup, spacer and lipid domain. They were used to prepare a series of lipidic formulations using GL67A composition as a guide, assuming that the parameters identified as optimal for GL67 would be similar for other related lipid-based formulations. Thus, GL67A was challenged with lipid formulations that, in most cases, combined a cationic lipid, a colipid (either DOPE or another), and DP5K. The possibility to prepare such formulations at high concentrations was first determined. Those for which this was feasible were then mixed with pDNA to obtain concentrated lipoplexes, which were mostly characterized with a charge ratio and a PEGylation ratio similar that of GL67A-based lipoplexes. Colloidally stable lipoplexes were then used for conducting aerosol delivery experiments in mice, with the aim to assess both safety and efficacy. Finally, the best lipidic formulation identified was used to conduct further complementary assays both in vitro and in vivo. Following this strategy, we identified a lead formulation characterized with properties capable of challenging those of GL67A under various experimental conditions (see below). A synopsis depicting the overall experimental plan with the main results obtained is available in Supplementary Materials.

### 3.2. Original Cationic Lipids Were Synthetized

**Scheme 1:**
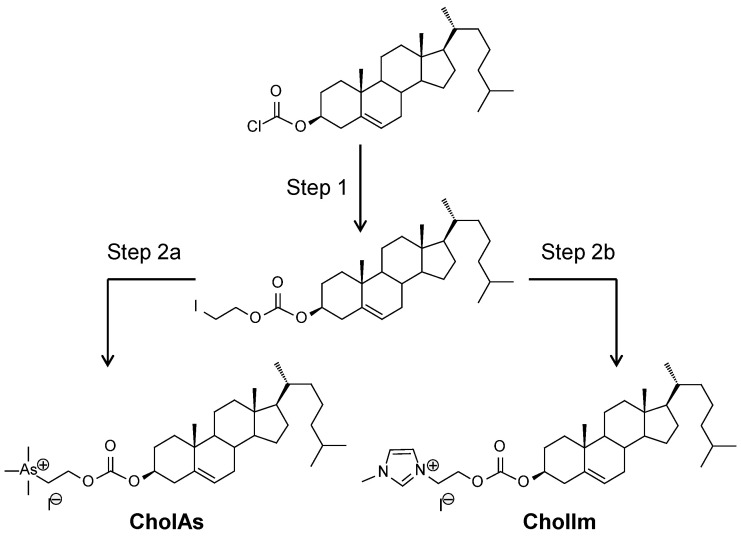
Two-step synthetic routes for obtaining the cholesterol-based trimethylarsonium cationic lipid CholAs and the cholesterol-based imidazolium cationic lipid CholIm. Step 1: 2-iodoethanol, Et_3_N, CH_2_Cl_2_, 0 °C to RT for 18 h (98% yield); Step 2a: Trimethylarsine, THF, 60 °C for 72 h (60% yield); Step 2b: *N*-methyl-1,3-imidazole, neat, 40 °C for 20 h (60% yield).

We first report three novel cationic lipids, namely CholAs, CholIm, and CholRi (Figure 1). Briefly, CholAs and CholIm were obtained following two-step synthetic routes (Scheme 1). First, cholesteryl chloroformate was reacted with iodoethanol to produce 2-iodoethylcholesteryl-carbonate. Next, the reaction of the latter intermediate with trimethylarsine on one hand or *N*-methyl-1,3-imidazole on the other made it possible to obtain CholAs or CholIm, respectively (both in 60% yield). See Supplementary Materials for more details about synthesis and characterizations.

### 3.3. Cationic Lipids and Colipids with Structural Diversity Were Considered

Eleven cationic lipids and five colipids were included in this study. They were selected due to their diverse chemical structures and previous investigations showing interesting physicochemical properties and biological activities (Table 1). The cationic lipids (Figure 1) were DOGB [23], DOPIm [27], DOPAs [20], DOSP [28,29], CholAs (this study), CholIm (this study), CholK [30,31,32], CholKB [30,31,32], CholP [29], CholRi [29], and CholT [33]. From a structural point of view (Table 1), these compounds can be distinguished according to their polar headgroup, consisting of either a monocationic permanent cation (trimethylarsonium in DOPAs and CholAs; ammonium in DOGB; imidazolium in DOPIm and CholIm); or a protonatable polycation (incorporating both primary and secondary amines in GL67, DOSP, CholP, CholK, CholT, CholKB, and CholRi). The lipidic domain was either two dialkyl (oleic, C18:1) chains (in DOPAs, DOGB, DOPIm, and DOSP) or a cholesterol moiety (in CholAs, CholIm, CholP, CholK, CholT, CholKB, CholRi, and GL67). These structural parts conferred an overall geometry more or less specific to each compound or group of compounds. Generally speaking, cationic lipids are also characterized by their lipophilic/hydrophilic balance, which can be estimated by calculating the partitioning value (hereafter termed cLogP). It is noteworthy that compared to the almost null (0.9) cLogP of GL67, the cationic lipids considered were predominantly either hydrophobic (cLogP between 4.5 and 14.2 for DOPAs, DOGB, DOPIm, CholIm, and CholAs) or hydrophilic (cLogP between −6.8 and −12.4 for DOSP, CholP, CholT, CholK, CholKB, and CholRi). The colipids (Figure 2), were DOPI [17], tetraether [23,34], diether [35], cholesterol, and DOPE. The latter can enhance the fusogenic property of lipidic assemblies [40,41], which may favor endosomal disruption during cell transfection, leading to a better release of pDNA into the cytoplasm [42]. Conversely, cholesterol can confer rigidity to liposomal assemblies [43]. DOPI is a lipophosphoramide incorporating an imidazole headgroup protonatable at acidic pH (lower than six); this feature is also presumably favorable for endosomal escape and it may explain that higher transfection efficiency can be obtained with DOPI compared to DOPE [17]. Tetraether is an archaeal bipolar lipid. In a previous study, this monolayer-forming lipid demonstrated contrasting effects for in vivo gene transfer depending on (i) the cationic lipid (DOPAs or DOGB) with which it was combined, and (ii) the delivery route (either intravenous or nasal instillation) used for administration in mice [23].

### 3.4. All Combinations of Lipidic Compounds Cannot Be Stably Formulated at High Concentrations 

The various abovementioned compounds (Figure 1 and Figure 2) were used in combination with the steric stabilizer DP5K to conceive a series of original lipidic formulations, based on GL67A composition (i.e., GL67/DOPE 1/2 DP5K, with GL67 = 15 mM and DP5K = 3.75 mg/mL) [10,15]. These formulations thus consisted of a cationic lipid used alone or combined with a colipid at a defined molar ratio; unless otherwise stated, following the hydration of the lipidic film formed by the latter, DP5K was added (post-insertion method) at various concentrations (ranging from 2.5 to 15 mg/mL). Table 2 and Table S1 provide information regarding the composition and physicochemical characterizations of the lipidic formulations performed at high cationic lipid concentrations (from 3.75 to 30 mM). Some of them did not yield homogeneous solutions, presumably because the compounds did not correctly assemble/mix together at the high concentrations needed for aerosol delivery. Following this screening procedure, we then particularly focused on a series of lipidic formulations (noted from F1 to F20), which were composed of relatively homogenous and small liposomes with clearly positive zeta potentials (Table 2).

### 3.5. All Lipidic Formulations Were Not Stable When Mixed with Highly Concentrated pDNA 

The lipidic formulations performed were then mixed with a pDNA, to examine the complexes obtained when prepared at the high concentrations required for subsequent aerosol experiments in mice. For this purpose, the assays were performed on a reduced scale (i.e., 1/100 of the volume needed to achieve an aerosol experiment in mice). In practice, 250 µg pDNA in 50 µL were gently mixed with 50 µL of a given lipidic formulation then left at RT for 15 min. Thanks to this protocol, we identified lipidic formulations that could be mixed with pDNA to form concentrated, colloidally-stable, and relatively homogenous (with a low polydispersity index) solutions (Table 3). By contrast, other mixtures formed aggregates or precipitates that could not be characterized (Table S2). Irrespective of the lipids combined, the colloidal stability of lipoplexes was clearly related to the amount of DP5K added into the lipidic formulation; in most instances, a PR (i.e., DP5K/pDNA mass ratio) lower than 1 was not sufficient to yield homogenous mixtures. It is noteworthy here that the GL67A + pDNA formulation was characterized by a PR of 0.75, which is thus slightly lower than the PR of our lipid + pDNA formulations (Table 3). The main lipidic formulations considered in the present study (F1 to F20, Table 2) were noted as “F# + L” or “F# + C”, the letters “L” and “C” referring to the luciferase-encoding (pGM144) and the CFTR-encoding (pCIK-CFTR) pDNA, respectively.

### 3.6. All Lipoplexes Showing Colloidal Stability Were Suitable for Delivery via Aerosol

The PARI LC PLUS nebulizer generated an aerosol for all the lipid + pDNA formulations evaluated, which could be inhaled by the mice placed inside the exposure box (Figure 3 and Figure S1). It is noteworthy that no problems occurred during nebulization (such as excessive bubbling or foaming) that would have affected it. Typically, the nebulization lasted between 30 and 45 min. While the pDNA, when used alone, was subjected to degradation due to the shear forces occurring during nebulization, it remained efficiently compacted and protected within most of the lipoplexes studied, whether the latter effectively reached the exposure box or did not leave the nebulizer reservoir (Figure S2A and data not shown). However, in some cases, pDNA degradation could be detected when assaying the lipoplexes taken from the nebulizer reservoir at the end of the nebulization process. When considering the physicochemical parameters of the lipoplexes studied before and after nebulization, the surface charge and size remained in the same range or slightly increased, particularly for complexes that remained in the nebulizer reservoir. This is shown, for example, with F18 + L and F20 + L (Figure S2B).

### 3.7. All the Aerosols Performed Were Safe for the Animals

In every instance, the formulation studied was well-tolerated by the mice, with no obvious clinical adverse effect being observed at any time of the experiment. During nebulization, mice remained active, moving freely inside the exposure box and breathing normally (e.g., showing no spasms). The mice usually rose on their hind feet under the aerosol coming from the conducting tube and falling into the box (Video), suggesting that they did not experience any irritation. Thereafter, and throughout the period during which the animals were observed, they behaved normally, without any signs of stress, pain, or discomfort; they did not lose weight but continued growing, with a growth curve similar to or only slightly delayed compared with the control, untreated mice. After euthanasia, the lungs always exhibited a normal appearance, both at early (24 h) and late (28 up to 400 days) time points after aerosol treatment (see below). Additional biochemical measurements performed on sera showed no elevation of ALT or AST, indicating the absence of injury to organs, especially the liver (data not shown). This finding was in accordance with previous studies performed using similar synthetic formulations that induced only transient and always reversible hepatotoxicity following intravenous delivery [22,23]. It is also noteworthy that in most cases, luciferase expression detected 24 h after treatment could be still detected for days thereafter, and until the end of the experiment. Thus, no significant loss of transgene expression occurred in any case, indicating that the lung-transfected cells were not adversely affected. Altogether, these results and those of earlier studies [12,13] support the generally excellent tolerability of aerosol-based non-viral gene therapy.

### 3.8. Aerosolized Formulations Demonstrated Diverse Abilities to Transfect Lungs In Vivo

**Figure 4:**
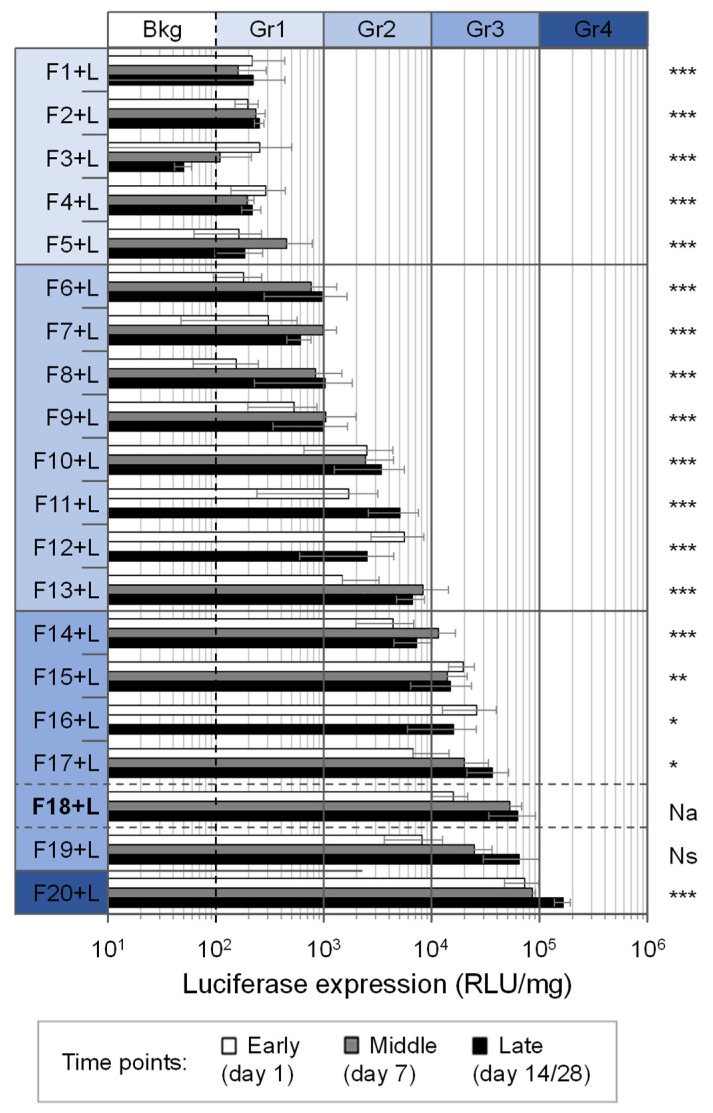
Luciferase activity in lung homogenates at three time points following aerosol treatment with various lipid + pGM144 formulations. Results correspond to means +/− SD, with *n* = 5 mice/group i.e., at each time point. The dashed vertical line specifies the luminescence background (Bkg, as determined with naive mice, ~10^2^ RLU/mg protein). Formulations are numbered from F1 to F20, according to the MLE (see Table 3 legend). Results for F11 + L, F12 + L, and F16 + L were not determined on day 7. The statistical significance determined when comparing the MLE of a given formulation with that of GL67A is indicated as follows: ***, *p*-value < 0.0005; **, *p*-value < 0.005; *, *p*-value < 0.05; Na, not applicable; Ns, non-significant.

Almost all of the aerosols evaluated transfected the lungs of the mice; 15 out of 20 aerosolized formulations yielded in vivo luciferase activity ≥ 10^3^ RLU/mg protein, i.e., at a level clearly above the background (10^2^ RLU/mg) determined using naive, untreated, mice (Figure 4). Overall, the luciferase levels measured in lung homogenates varied over four logs of amplitude. For the formulations demonstrating some efficiency, the kinetics of luciferase expression could vary, such that transgene expression either increased, slightly decreased, or remained nearly stable during the 14 to 28 days of the experiment. Based on the maximal luciferase expression (MLE) being reached at 1, 7, 14 or 28 days after aerosol treatment, the various formulations were classified into four groups: ineffective, slightly efficient, efficient, and highly efficient (Table 3). Besides GL67A, formulations prepared with either CholP, CholT, CholK, or CholKB (but not CholRi or any other cationic lipid) mediated luciferase expression > 10^4^ RLU/mg. Among all these, only the CholP-based formulation was able to reach >10^5^ RLU/mg. The highly sensitive luciferase activity measurements indicated that the latter outperformed GL67A with significantly (*p*-value = 0.0079) better (two to five times higher) transgene expression at all the time points of the experiment. Altogether, the results obtained allowed the delineation of a series of structure/activity relationships (see Figure S3 and discussion below).

### 3.9. Further Studies Detailed the Interest of CholP/DOPE for Aerosol Lung Gene Delivery

Given that CholP was identified herein as the most efficient cationic lipid for aerosol lung gene delivery in mice, additional studies were conducted focusing on formulations incorporating this compound.

**Figure 5:**
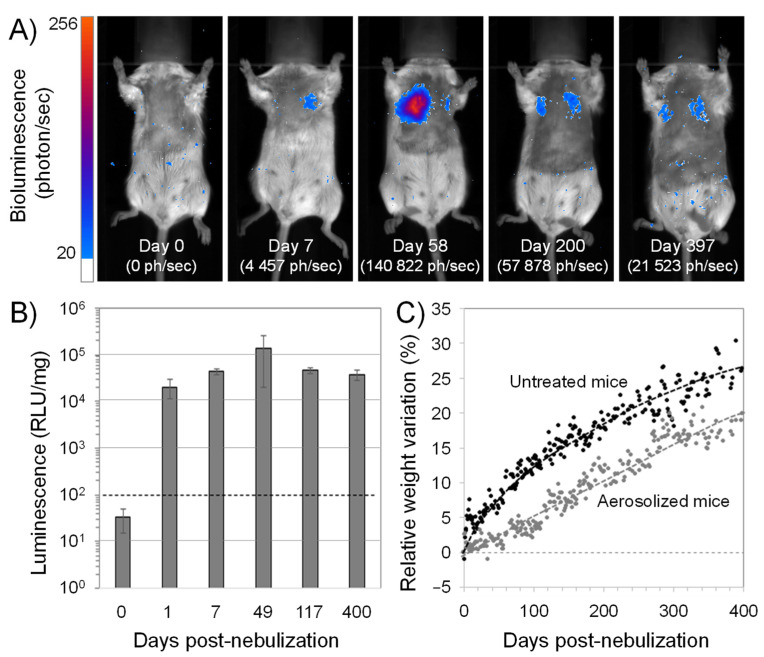
Long-term, longitudinal follow-up of mice following treatment with an aerosol of F20 + L (((CholP/DOPE 1/2 DP5K) + pGM144) CR2 PR2). Panel (**A**) shows representative images of in vivo bioluminescence captured before treatment and then 7, 58, 200, and 397 days after treatment. The color scale denotes bioluminescence intensity; it is set to display only signals above the background. For each mouse, luminescence intensity is indicated. See Figure S4 for more pictures and information. In panel (**B**), luciferase expression was determined in lung homogenates collected before treatment and then 1, 7, 49, 117, and 400 days after treatment (mean +/− SD with *n* = 3–5 mice at each time point). The dashed horizontal line specifies the positivity threshold in luminescence. Panel (**C**) recapitulates the body weight variations of the mice during the experiment; each dot corresponds to the mean in either a control group (*n* = 5 mice) or a treated group (*n* = 3–20 mice).

**Figure 6:**
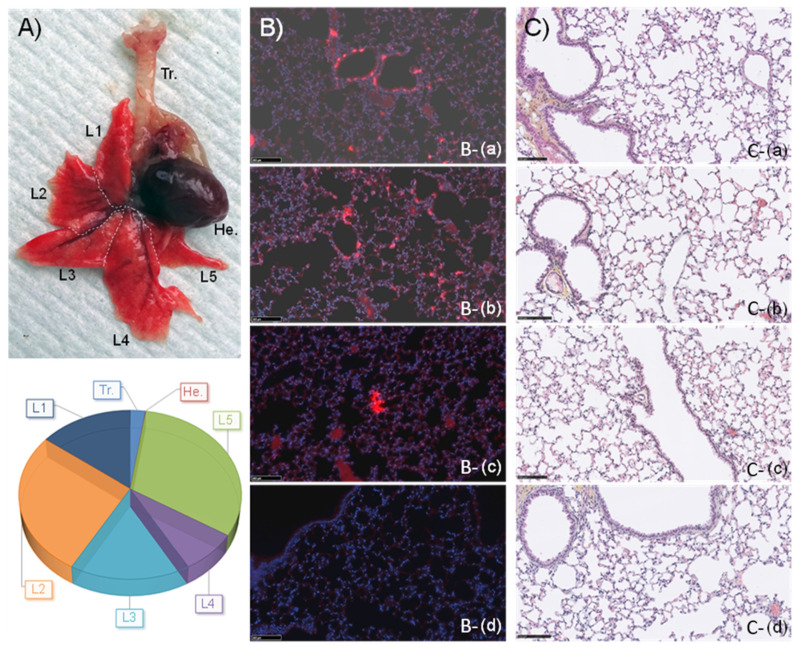
Luciferase expression in lungs 7 days after treatment of mice with an aerosol of F20 + L ([(CholP/DOPE 1/2 DP5K) + pGM144] CR2 PR2). Panel (**A**) illustrates the distribution of luminescence in lung lobes (L1–L5), heart (He.), and trachea (Tr.). Dashed white lines are used to help individualize the different lobes. The heart was used as a negative control for transfection. Panel (**B**) corresponds to immunohistochemistry analyses for luciferase detection in treated (**Ba**–**Bc**) compared to untreated (**Bd**) lungs. Luciferase-expressing cells and nuclei are stained with Rhodamine (red) and DAPI (blue), respectively. Panel (**C**) corresponds to hematoxylin and eosin (H&E) stain of lung sections from the same mice. The scale bar in every image is 100 µm. See Figure S5 for more images.

(1) Firstly, a long-term longitudinal follow-up was performed with mice treated with an aerosol of [(CholP/DOPE 1/2 DP5K) + pGM144] CR2 PR2. Seven days after treatment, luminescence was strong enough (i.e., ≥10^4^ RLU/mg of protein [25]) to be detected from living animals via whole-body animal imaging (Figure 5A and Figure S4). The luminescence was collected from the surface of the chest area, forming a single or two distinct patches likely originating from the right and the left lung lobes, respectively. BLI was further detailed with measurements performed on organs harvested at several time points (i.e., 1, 7, 49, 117, and 400 days after aerosol treatment), confirming that the reporter gene expression could be detected in the lungs but not in any other body part (Figure 5B and data not shown). Furthermore, BLI as well as luminescence measurements in lung homogenates both showed that transgene expression increased after treatment, reached a peak (>10^5^ RLU/mg) about 50 days later, and then remained stable (at about 5 × 10^4^ RLU/mg) until the end of the experiment. Thus, a single aerosol treatment with this formulation was able to mediate sustained luciferase transgene expression in murine lungs for as long as 400 days (a duration that may be even longer, potentially reaching the lifetime of BALB/c mice i.e., about 2 years; for ethical reasons, it was not possible to monitor the animals for a longer duration). During this period of time, the animals continued growing; they showed on average a slight but persistent growth delay compared with the untreated mice, which could not be explained in this study (Figure 5C).

(2) Seven days after aerosol delivery, luciferase expression was detectable in every lung lobe and also, to a lesser extent, in the trachea. By contrast, no luminescence could be measured in the heart (Figure 6A). Immunohistochemical analysis revealed luciferase-positive epithelial cells distributed in the lungs and sometimes grouped as clusters of intense positive foci (Figure 6B). H&E staining showed that the microanatomy of lungs was similar in treated and untreated mice lungs (Figure 6C). Thus, CholP/DOPE 1/2 yielded a widespread transgene (luciferase) expression in the lungs.

(3) CholP/DOPE 1/2 DP5K was also used to deliver via aerosol a pDNA encoding a human CFTR transgene, to CFBE cells and Cftr−/− mice concomitantly (Figure S6). As for cells, high expression levels of the transgene-encoded hCFTR protein were detected with no adverse cytotoxic effects observed (Figure S6C). In the mice, immunohistochemistry performed on lungs harvested 24 h after aerosol revealed slightly positive transfected cells. However, no difference in terms of CFTR expression could be found when comparing the GL67A and CholP-based formulations. In every case, histological examinations of lungs revealed a normal tissue architecture without tissue injury, fibrosis, or macrophage infiltration (Figure S6D). 

(4) CholP/DOPE 1/2 DP5K and GL67A were assayed under in vitro experimental conditions closer to CF mucus airways. For this purpose, the lipoplexes tested in animals (i.e., at CR2, but also at two other CR, i.e., CR1 and CR4) were mixed with competitive components typically found in CF mucus (i.e., linear DNA, albumin or mucine) before being used to transfect CFBE cells. In almost every case, the cell viability was found to be preserved (i.e., ≥80%). In agreement with previous evaluations [11], GL67A showed resistance up to very high concentrations of mucus. CholP-based lipoplexes were also able to transfect CFBE cells, although with less efficiency and with more sensitivity to mucus components, particularly to mucine and linear DNA (Figure S7). For both lipidic systems, better resistances could be measured when assayed at CR higher than 1. Beyond these in vitro tests performed by deposition, other experiments performed via aerosol demonstrated similar trends (not shown).

(5) The experiments using clinical strains of *Staphylococcus aureus* and *Pseudomonas aeruginosa* (which are two bacterial species typically found in the lungs of CF patients) showed that the CholP/DOPE formulation did not exhibit any antibacterial effects (at least up to 1024 µM) (Figure S8); the same results were obtained when using the CholK, DOSP, and DOSK formulations (data not shown). Importantly, while the bacteria developed resistance when exposed for several passages to sub-inhibitory doses of either Kanamycin or Paromomycin, their susceptibility to these antibiotics remained unchanged after repeated exposure to a high dose (100 µM) of the corresponding aminoglycoside-based cationic lipids (Figure S8). 

(6) Lastly, the CholP/DOPE 1/2 DP5K liposomes (as well as other concentrated formulations of aminoglycoside-based cationic lipids) remained stable during storage at 4 °C for several months, showing no precipitation detectable by eye and no noticeable variations in their physicochemical properties (Table S3).

## 4. Discussion

Currently, GL67A is still the lead product for clinical CF non-viral gene therapy. Since the seminal work by Lee et al., published in 1996, reporting this lipidic formulation for the first time [10], no obvious alternative has been found or clearly shown to challenge its efficacy. This may be due to the fact that different experimental procedures and/or different reference compounds have been used, preventing proper comparisons. However, many non-viral delivery systems have been compared with GL67A for CF gene therapy in preclinical studies. This includes cationic (e.g., polyethylenimine (PEI) [12]) or neutral (e.g., tetra-functional block copolymer [44,45]) polymers and solid nanoparticles (e.g., carbon dot nano-carriers [46]). In these studies, instillation in the mouse trachea was used as the delivery method to evaluate gene delivery efficiency. Despite these original compounds exhibiting interesting potencies compared with GL67A, delivery in the form of an aerosol to target the airway epithelium in vivo was not assessed. More recently, Suk et al. reported highly compacted biodegradable DNA nanoparticles capable of overcoming the mucus barrier for inhaled lung gene therapy [47]. These authors showed that poly(β-amino-esters) were able to provide higher transgene expression in murine lungs compared with other non-viral platforms including polylysine and PEI [12,47,48,49]. However, it is noticeable that the delivery route was local inhalation (using a microsprayer rather than a clinical nebulizer) and thus this synthetic carrier was not evaluated under clinically relevant conditions.

One important restriction on systematic evaluation following nebulization to mice is the large quantity of materials needed. In order to deliver sufficient amounts of a given formulation for inhalation by the animals, an aerosol experiment typically requires several tens of milligrams of pDNA [36,50]. In our own study, 25 mg pDNA were used for each evaluation. This is in the same range as for dosing a single human subject (e.g., each patient enrolled in the latest clinical trial conducted by the UK CF Gene Therapy Consortium received 13.3 mg pDNA every month [4]). This highlights the importance of being able to produce new formulations at a scale suitable for clinical use. Herein, all the cationic lipids and colipids (Figure 1 and Figure 2) were obtained in gram-scale quantities with high purity levels, following protocols either previously reported [17,20,23,27,28,29,30,31,32,33,34,35] or modified for this study (see supporting information). The large quantities needed for a single aerosol experiment (as described herein) may explain why most non-viral delivery systems have been compared using delivery methods inferior to nebulization (i.e., systemic injection, intra-nasal deposition, intra-tracheal instillation, or intra-tracheal inhalation), which all require significantly lower amounts of materials [21,23,25]. However, these alternative dosing methods exhibit inherent limitations, since the results cannot be directly translated to the aerosolization setting [21,25,26]. Hence, nebulization under “clinical trial-like conditions” remains the preferred preclinical screening method to assess the potency of gene delivery systems designed for targeting the airways. Compared with the whole-body animal exposure protocol used in our study (Figure 3 and Figure S1), other experimental (e.g., “snout only” exposure) set-ups may allow a more direct delivery to the airways while limiting the loss of material; however, such procedures do not allow substantial reductions in the amount of pDNA needed and the pDNA/mouse ratio remains in the same range as in the present study [14]. Furthermore, we observed that the use of classic (submerged) cell culture instead of mice was only poorly-predictive of the in vivo efficacy. Although all the formulations tested in this study showed some ability to deliver a luciferase transgene to cell lines in vitro (Figure S9), only some were efficient at the transfection of lung epithelial cells in vivo (Figure 4). For these reasons, the use of animals still remains inescapable. 

Based on luciferase reporter gene expression in the lungs, the various formulations aerosolized to animals were not equivalent for gene delivery (Figure 4 and Table 3); comparing luminescence intensities in lung homogenates helped the delineation of a series of structure/activity relationships (Figure S3). (i) As regards the cationic lipid itself, the polar headgroup and the lipidic domain play obvious critical roles. Irrespective of other structural features, the formulations prepared with a multivalent cationic lipid (bearing several protonatable amines) were in almost every instance significantly more efficient than the formulations produced with a monocationic lipid (exhibiting a single permanent cation) (Figure S3Da). It is noteworthy that, for reaching a given charge ratio with pDNA, lower molar quantities are needed for the former than for the latter. Furthermore, cationic lipids incorporating a cholesterol moiety were more efficient than those bearing two oleic chains (Figure S3Db). This was obvious when comparing the transfection efficiencies of formulations incorporating CholP (F20 + L) or DOSP (F6 + L). Furthermore, the cationic headgroup and the lipidic domain determine a hydrophilic/lipophilic balance that can be estimated with the calculated (predicted) cLogP value. It is noteworthy that the formulations incorporating a cationic lipid exhibiting cLogP < 1 (i.e., which were predominantly hydrophilic) were significantly more efficient than those incorporating a cationic lipid featuring cLogP > 1 (Figure S3Dc). In the present study, the ((CholP/DOPE 1/2 DP5K) + pGM144) CR2 PR2 formulation (F20 + L) was, at each time point (i.e., on day 1, 7 and 28), significantly (*p*-value = 0.0079) more (two- to five-fold) efficient than GL67A (F18 + L) (Figure S3Dd). Comparing the chemical structure of GL67 with that of CholP is informative as it verifies some assumptions relating to the potential role of all three domains of the cationic lipid [10]. It was proposed that the high efficiency of GL67 was related to the “T-shape configuration” of its headgroup. The T-shaped polyamine could indeed promote more productive interactions with pDNA; this headgroup may also resemble a cognate ligand for a cell-surface receptor, thereby facilitating attachment and entry into target cells. The secondary amine at the junction between the headgroup and the spacer may also participate in pDNA interaction and, later, in the endosomal escape during intracellular trafficking. GL67-derivatives incorporating dialkyl chains instead of a cholesterol moiety were found to be significantly less efficient than the canonical GL67 [10]. It is noteworthy that the results obtained with the lipidic derivatives used in the present study supported similar conclusions. The cholesterol derivative of paromomycin CholP exhibited a T-shape conformation and multiple primary amines protonatable at physiological pH. The natural affinity of aminoglycosides such as paromomycin for nucleic acids further argues for a dominant role of the electrostatic and structural interaction between the headgroup and pDNA. (ii) The charge ratio is another key parameter. Considering the DOSP/DOPI 1/1 formulation, lipid/pDNA complexes formed at CR2 (F10 + L) were found to be more efficient than those formed at CR1 (F3 + L) or at CR4 (F9 + L), thus again showing a convergence criteria with GL67A lipoplexes (Figure S3De). (iii) Besides the cationic lipid, the colipid is also determinant, depending on both its chemical structure and its proportion in mixture with the cationic lipid. For instance, CholP/DOPE 1/1 (F15 + L) was found to be somewhat less efficient than CholP/DOPE 1/2 (F20 + L), illustrating the importance of the colipid proportion (Figure S3Df). CholP/DOPE 1/1 (F15 + L) and CholP/DOPI 1/1 (F16 + L) reached transfection efficiencies of the same order of magnitude as that of GL67A (F18 + L) (Figure S3Dg). It was noticeable that CholP/Diether 1/2 (F11 + L) was significantly (*p*-value = 0.0079) less (32 to 43 times) efficient than CholP/DOPE 1/2 (F20 + L). Another formulation of CholP with tetraether would have provided complementary and interesting insights into the role of that particular colipid. Altogether, these results highlight the transfection potential of the cholesterol aminoglycoside derivative CholP, as well as the usefulness of a colipid such as DOPE or DOPI for enhancing its activity. It is noticeable that both GL67 and CholP are multivalent protonatable cholesterol derivatives combined in F18 and F20 with a fatty acid-based colipid. The cholesterol ring may provide some rigidity to the pDNA complexes, allowing them to resist shear forces during aerosolization. Furthermore, a colipid such as DOPE (or possibly also DOPI) may provide the pDNA complexes with some fusogenicity, which is particularly useful for endosomal escape. Conversely, the inefficiency of the CholAs formulation (F1 + L) may have been due to the fact that it is a monovalent permanent cation and it was not combined with a colipid such as DOPE. In an attempt to better understand the importance of the cholesterol moiety for aerosol delivery, we used cholesterol as a colipid in combination with DOSP as a cationic lipid; unfortunately, DOSP/Chol could not be formulated (i.e., correctly assembled at the high concentration needed). The results obtained with the DOPIm/DOPI 1/1 formulation (F12 + L) were also informative; this formulation yielded quite good transfection efficiencies, despite DOPIm and DOPI being two lipids incorporating aliphatic (oleyl) chains—they do not contain a cholesterol moiety—and DOPIm incorporates a monovalent permanent cationic headgroup. The combination of these two lipophosphoramides (which are highly similar structurally, differing only in that the DOPIm headgroup is based on an imidazolium group, whereas DOPI harbors an imidazole group) was previously found to be quite efficient for gene transfection in general [17,51]; this may be due to the protonability of the imidazole group at acidic endosomal pH with a proton sponge effect and an increased fusogenicity. 

Aminoglycoside-based cationic lipids constitute a class of poly-functional synthetic compounds [52,53] with great potential for gene transfection in vitro as well as in vivo [29,54,55,56,57]. They were shown to be efficient in the intracellular delivery of siRNA [28], miRNA [58], mRNA [32], and functional proteins [59,60]. The role of the three domains (headgroup, spacer, and lipidic domain) of these cationic lipids was outlined. The modulation of the lamellar phase of their assemblies with pDNA was shown to facilitate the endosomal release step during intracellular trafficking [61,62]. The selective condensation of DNA by aminoglycoside antibiotics has also been reported [63]. Generally speaking, a compromise is needed between the stability of complexes outside of cells (required for efficient compaction and protection of pDNA, during delivery and trafficking) and their instability once inside the cells (for a productive release of the pDNA cargo) [22,64]. It is noteworthy that, although they are efficient at compacting and protecting pDNA, both GL67A and lipidic aminoglycoside derivative-based formulations apparently formed lipoplexes in which pDNA, was not as strongly compacted as in formulations formed with cationic lipids bearing a monovalent permanent cation. Since the modification of the constitutive subunits of aminoglycoside-based gene carriers has been associated with substantial changes in transfection abilities [56], cholesterol-based cationic lipids incorporating other aminoglycoside headgroups were also evaluated in this study (Figure 1). Each was used to prepare a formulation with the same composition as that of CholP/DOPE 1/2 DP5K (F20). Except for CholRi, all these additional lipidic aminoglycoside derivative-based formulations mixed with pGM144 were able to reach luciferase expression levels above 10^4^ RLU/mg protein (Figure 4), thus demonstrating the general potential of cholesterol-based aminoglycosides for aerosol gene delivery. In every case, the most efficient formulation was ((CholP/DOPE 1/2 DP5K) + pGM144) CR2 PR2 (F20 + L), mediating significantly higher luciferase activity in the lungs than all the others (Figure S3Dh). Twenty-eight days after aerosol, luciferase expression measured with CholP was indeed on average 3, 5, 23, and 25 times higher than that of CholT, CholK, CholKB, and CholRi, respectively. Such differences can be ascribed to variations in the chemical structure of aminoglycoside headgroups. First, except for CholRi, CholP exhibits a T-shape configuration with four ring subunits (including a ribose) whereas CholT, CholK, and CholKB all feature a linear sequence of three ring subunits (without any ribose). Second, slight chemical variations (involving a single moiety) can impact gene delivery efficiency, as shown by the comparison of luciferase expression levels measured with CholT versus CholK (the latter bearing an amino group in place of a hydroxyl) on one hand and with CholT versus CholKB (the latter bearing an additional hydroxyl group) on the other (Figure S3). Increasing the number of amino functions at the expense of hydroxyl functions was reported to impact on the efficiency of the resulting vectors [29]. As for CholRi, its peculiar structure (consisting of three ring subunits, including a ribose) was found to be detrimental for aerosol gene delivery. Taken altogether, our results are consistent with earlier studies reporting that various lipidic aminoglycoside derivatives can yield very different transfection activities (Figure 4 and Figure S9) [29]. In these previous studies, CholP was not identified as the most efficient, a finding highlighting that each application may require specific reagents. In summary, CholP might exhibit a series of advantageous structural features, which render it highly suitable for aerosol gene delivery in murine lungs.

Persistent in vivo transgene expression was measured for 400 days after a single aerosol treatment with a CholP-based formulation (F20 + L), which could potentially last for the lifetime of the animals (Figure 5 and Figure S4). To our knowledge, this has not been published previously for a non-viral formulation (however, a similar duration of in vivo expression has been observed with GL67A [D. Gill, personal unpublished data]). At present, the reason for such enduring transgene expression remains unclear; it may result from a combination of parameters related to the delivery system and pDNA used as well the cell type(s) transfected. (i) In terms of the lipidic formulation used, this result further suggests that it is characterized with a good safety profile since transfected cells can persist for a long time with efficient transgene expression. (ii) With respect to the DNA delivered, pGM144 exhibits some properties that are involved in high and sustained transgene expression in vivo [36]. When GL67A and CholP were used to deliver the CFTR-encoding pDNA pCIK-CFTR, both formulations mediated similar levels of CFTR protein, whether in cells or in CF-model mice (Figure S6). However, while strong levels of CFTR protein were detected in vitro, weaker expression levels were obtained in vivo. Compared with pGM144, which is completely free of CpG dinucleotides, pCIK-CFTR contains many CpGs and is thus not optimal for in vivo studies. A more suitable pDNA would be the CpG-free CFTR-encoding pGM169 used in the latest clinical trial conducted by the UK CF Gene Therapy Consortium [4]. However, irrespective of the pDNA used, prolonged transgene expression is quite unexpected since plasmid DNA supposedly remains as an episome i.e., it does not insert in the host genome. This suggests the contribution of other parameter(s) related to the cell type(s) transfected. (iii) As regards the latter, it was not identified herein; this identification should take place in an ongoing study. However, some hypotheses can be established considering the known persistence of lung epithelial cells in vivo. According to Rawlins and Hogan, ciliated airway epithelial cells in murine lungs are a terminally differentiated population, with an average half-life of 17 months [65]. As reported previously with lentiviral vectors, basal cells may play a role in persistent airway gene expression [66]. It has been suggested that long-term foreign gene expression can be obtained from episomal pDNA if the target cell is post-mitotic or slowly mitotic and no immune reaction against the transgene-derived protein is elicited [67,68,69]. Finally, it is worth noting that luminescence expression continued to increase several days after aerosol treatment (Table 3, Figure 4, Figure 5 and Figure S4) and strong localized luminescence foci could be detected in the lungs of mice (Figure 6B). From these findings, a prolonged in situ release from some “lipoplex reservoirs” can be hypothesized, which should also be further investigated in another study.

To further determine whether CholP may be suitable for use in CF gene therapy, additional experiments were conducted under experimental conditions more similar to the CF airways. (i) It has been shown previously that mucus components forming the viscous secretions lining the upper airways and bronchi of CF patients may interact with lipoplexes, resulting in a loss of their transfection activities [11]. Thus, gene transfections were performed in the presence of linear DNA, albumin, or mucine that are typically found in CF airways. Altogether, the results obtained from a dose escalation study (Figure S7) suggest that, although they are more sensitive than GL67A, CholP-based lipoplexes may retain some transfection efficiency under CF relevant conditions. This also suggests the requirement to further optimize CholP formulation, especially as regards its content in DP5K, and to investigate more complex cell culture models [70,71]. (ii) Considering bacterial infections that occur in the airways of CF patients, additional antibacterial properties of cationic lipids [18] or polymers [72] might be useful for CF lung gene therapy applications. Therefore, lipidic aminoglycoside derivatives have been designed so that in addition to gene transfer activity, they could also display (i) biocide effects and (ii) premature stop codon read-through activity. As for the latter, it is noteworthy that aminoglycosides have been outperformed by PTC124 (Ataluren^®^), which was previously used in the clinical setting [73]. Considering GL67 cationic lipid, such additional activities have never been reported; and no antibacterial effect was detected with GL67A formulation in our study (data not shown). Contrary to kanamycin, tobramycin, and paromomycin, we found that lipidic derivatives of corresponding antibiotics (bearing either a cholesterol moiety or two aliphatic chains) displayed no activity towards various bacterial strains (*S. aureus*, *Escherichia coli*, and *P. aeruginosa*; Figure S8 and data not shown). Thus, increasing the hydrophobicity of these antibiotics leads to the loss of their antibacterial effect (possibly because diffusion through bacterial cell wall and/or interaction with bacterial ribosomal RNA targets are altered/prevented, due to steric hindrances). Consequently, these lipidic compounds may not be captured (titrated) by bacterial cells. Furthermore, this finding indicates that the formulations evaluated did not incorporate any significant traces of free antibiotics, emphasizing the quality of the chemistry protocol used for the synthesis and purification of these compounds. Besides, although they are devoid of antibacterial activity, we wondered whether lipidic aminoglycoside derivatives might participate in the emergence of multi-resistant strains of bacteria found in the lungs of CF patients. This hypothesis could not be ruled out since bacteria may be able to metabolize and degrade such compounds in different ways, releasing active aminoglycoside units at sub-inhibitory concentrations. To address this potential concern, we used both Gram-positive and Gram-negative clinical strains susceptible to paromomycin and kanamycin. The study showed that the use of CholP or CholK should be safe in that regard, since the susceptibility of *S. aureus* and *P. aeruginosa* to both paromomycin and kanamycin remained unchanged after repeated incubations with a high dose of cholesterol derivative of the corresponding antibiotics (Figure S8). In addition, contrary to tobramycin (which is routinely used as a solution for inhalation (Tobi^®^) to treat bacterial lung infections [74]), it is noteworthy that paromomycin is not commonly used for treating CF patients. Altogether, CholP appears as a potent alternative to GL67 for aerosol lung gene therapy, the use of which would not interfere with other treatments offered to CF patients.

## 5. Conclusions

The development of novel non-viral gene therapy vectors and formulations with clinical relevance is still required, but due to the relatively limited throughput of in vivo preclinical screening and the costly large quantities of materials needed, this remains a substantial challenge. In this study, we conducted a rational screening of a wide panel of formulations incorporating diverse lipidic structures either previously documented or described here for the first time. This allowed us to design formulations at least as efficient as GL67A and to unveil structure/activity relationships determining the efficiency of aerosol lung gene delivery. As regards the cationic lipid itself, multivalence, protonability, and a cholesterol moiety were identified as key structural features. On the other hand, the combination of the latter with an appropriate colipid at an adequate molar ratio showed potential to improve activities. This suggests the need to investigate the transfection efficiency of other cationic lipids in combination with appropriate colipids as well as alternative strategies based on other types of vectors. In particular, studying the inclusion of tetraether (rather than cholesterol) to provide rigidity to the complexes and assessing ternary formulations should be considered. Besides cationic lipids, cationic polymers evaluated under similar experimental conditions have also demonstrated promising gene delivery efficiency in the lungs of mice [50,75]. Combined synthetic systems consisting of a pDNA core condensed with PEI and encapsulated within a liposome-based formulation (i.e., lipo-polyplexes) are also under investigation; this may lead to the design of improved next-generation hybrid non-viral gene delivery systems capable of outperforming GL67A. Complementary evaluations (including transmission electron microscopy performed prior to and after nebulization) could also provide key insights into the parameters governing transfection efficiency. Importantly, these approaches should clearly indicate whether much higher transfection levels can be reached by using synthetic (lipid-based) nanocarriers; this is not obvious given the convergence observed between the vectors/formulations yielding the best results. In conclusion, this study further demonstrates the versatility of aminoglycoside-based cationic lipids and invites their evaluation for the delivery of other payloads relevant to CF gene therapy, such as CFTR-encoding mRNA and CRISPR/cas systems.

## Figures and Tables

**Table 1:** Structural features of the cationic lipids investigated.

#	CL	Counter Ion	MW ^1^	Geo.^2^	Headgroup	Z^+ 3^	Lipid Domain	cLogP ^4^	Reference
1	DOPAs	1 I^−^	872	C	Trimethyl-arsonium	Pc (1)	(C18:1)_2_	14.2	[20]
2	DOGB	1 Cl^−^	756	C	Glycine betaine	Pc (1)	(C18:1)_2_	8.5	[23]
3	DOPIm	1 Br^−^	787	C	Imidazolium	Pc (1)	(C18:1)_2_	10.1	[27]
4	DOSP	4 CF_3_COO^−^	1672	T	Paromomycin	Pa (4)	(C18:1)_2_	−6.8	[28]
5	CholAs	1 I^−^	705	L	Trimethyl-arsonium	Pc (1)	Chol	9.2	This study
6	CholIm	1 I^−^	620	L	Imidazolium	Pc (1)	Chol	4.5	This study
7	CholP	4 CF_3_COO^−^	1484	T	Paromomycin	Pa (4)	Chol	−12.4	[29]
8	CholT	4 CF_3_COO^−^	1336	L	Tobramycin	Pa (4)	Chol	−10.5	[33]
9	CholK	3 CF_3_COO^−^	1239	L	Kanamycin	Pa (3)	Chol	−8.1	[30,31,32]
10	CholKB	4 CF_3_COO^−^	1352	L	Kanamycin B	Pa (4)	Chol	−11.2	[30,31,32]
11	CholRi	3 CF_3_COO^−^	1209	T	Ribostamycin	Pa (3)	Chol	−7.5	[29]
12	GL67	2 Cl^−^	629	T	Spermine	Pa (2)	Chol	0.9	[10]

^1^ Molecular weight in g/mol; ^2^ geometry (C, conical; T, T-shape; L, linear); ^3^ protonatable amine (Pa) or permanent cation (Pc) in headgroup (number of positive charge at physiological pH 7.4); ^4^ calculated partitioning value (average value of octanol-water portioning coefficient) of ionic species, as determined using MarvinSketch 5.11.5 (Chemaxon).

**Table 2:** Composition and physicochemical characterizations of liposomes.

Lipidic Formulation Composition						Physicochemistry		
F#	CL	[CL] ^1^	Colipid(s) ^2^	MR ^3^	[DP5K] ^4^	Size ^5^	PdI ^6^	Zeta ^7^
F1	CholAs	15.0	None	Na	5	178	0.20	+50
F2	CholIm	30.0	DOPE	3/2	10	108	0.21	+50
F3	DOSP	3.7	DOPI	1/1	5	309	0.52	Nd
F4	DOPIm	30.0	Chol/DOPE	1/1/1	10	353	0.51	+50
F5	DOPAs	30.0	None	Na	5	136	0.26	+69
F6	DOSP	7.5	DOPE	1/1	10	254	0.42	Nd
F7	DOSP	7.5	Tetraether	5/1	10	117	0.25	+54
F8	DOGB	30.0	Tetraether	10/1	5	190	0.32	+28
F9	DOSP	15.0	DOPI	1/1	10	126	0.20	+53
F10	DOSP	7.5	DOPI	1/1	5	92	0.26	+67
F11	CholP	7.5	Diether	1/2	10	220	0.46	Nd
F12	DOPIm	30.0	DOPI	1/1	5	135	0.22	+38
F13	CholRi	10.0	DOPE	1/2	10	Nd	Nd	Nd
F14	CholKB	7.5	DOPE	1/2	10	Nd	Nd	Nd
F15	CholP	7.5	DOPE	1/1	10	143	0.29	+53
F16	CholP	7.5	DOPI	1/1	10	172	0.31	+57
F17	CholK	10.0	DOPE	1/2	10	Nd	Nd	Nd
F18	GL67	15.0	DOPE	1/2	5	294	0.27	+55
F19	CholT	7.5	DOPE	1/2	10	Nd	Nd	Nd
F20	CholP	7.5	DOPE	1/2	10	256	0.12	Nd

^1^ In mM; ^2^ in addition to DP5K; ^3^ cationic lipid/colipid(s) molar ratio; ^4^ in mg/mL; ^5^ in nm; ^6^ polydispersity index; ^7^ in mV; Nd, not determined; Na, not applicable. See Table S1 for results obtained with other formulations performed.

**Table 3:** Composition, physicochemical characterizations, and in vivo evaluation of lipoplexes.

Lipid + pDNA Formulation Composition						Physicochemistry			In Vivo Evaluation ^9^			
F# + L ^1^	CL	Colipid(s) ^2^	MR ^3^	PR ^4^	CR ^5^	Size ^6^	PdI ^7^	Zeta ^8^	D1	D7	D14/28	Group
F1 + L	CholAs	None	Na	1	1	325	0.36	+37	1	1	1	Gr1
F2 + L	CholIm	DOPE	3/2	2	2	160	0.29	+22	1	1	1	Gr1
F3 + L	DOSP	DOPI	1/1	1	1	307	0.52	Nd	5	2	1	Gr1
F4 + L	DOPIm	Chol/DOPE	1/1/1	2	2	197	0.18	+21	1	1	1	Gr1
F5 + L	DOPAs	None	Na	1	2	245	0.50	+38	1	3	1	Gr1
F6 + L	DOSP	DOPE	1/1	2	2	289	0.36	Nd	1	4	5	Gr2
F7 + L	DOSP	Tetraether	5/1	2	2	224	0.63	Nd	1	3	2	Gr2
F8 + L	DOGB	Tetraether	10/1	1	2	190	0.32	+28	1	5	7	Gr2
F9 + L	DOSP	DOPI	1/1	2	4	481	0.76	+1	1	2	2	Gr2
F10 + L	DOSP	DOPI	1/1	1	2	153	0.29	+5	1	1	1	Gr2
F11 + L	CholP	Diether	1/2	2	2	193	0.39	Nd	1	Nd	3	Gr2
F12 + L	DOPIm	DOPI	1/1	1	2	192	0.33	+43	2	Nd	1	Gr2
F13 + L	CholRi	DOPE	1/2	2	2	Nd	Nd	Nd	1	6	4	Gr2
F14 + L	CholKB	DOPE	1/2	2	2	Nd	Nd	Nd	1	3	2	Gr3
F15 + L	CholP	DOPE	1/1	2	2	307	0.52	Nd	1	1	1	Gr3
F16 + L	CholP	DOPI	1/1	2	2	259	0.33	Nd	2	Nd	1	Gr3
F17 + L	CholK	DOPE	1/2	2	2	Nd	Nd	Nd	1	3	5	Gr3
F18 + L	GL67	DOPE	1/2	1	2	262	0.22	+30	1	3	4	Gr3
F19 + L	CholT	DOPE	1/2	2	2	Nd	Nd	Nd	1	3	8	Gr3
F20 + L	CholP	DOPE	1/2	2	2	168	0.27	Nd	1	1	2	Gr4

^1^ Lipid formulation (F#) mixed with the luciferase-encoding pDNA pGM144 (L); ^2^ in addition to DP5K; ^3^ cationic lipid/colipid(s) molar ratio; ^4^ DP5K/pDNA mass ratio; ^5^ cationic lipid/pDNA charge ratio; ^6^ in nm; ^7^ polydispersity index; ^8^ in mV; ^9^ relative lung reporter gene expression on day 1 (D1), 7 (D7) and 14/28 (D28) (the minimal luciferase expression being given the value of 1) and classification according to the maximal luciferase expression (MLE) reached (on average on day 1, 7, or 14/28), as follows: Gr1 (inefficient), MLE ≤ 10^3^; Gr2 (slightly efficient), MLE ≤ 10^4^; Gr3 (efficient), MLE ≤ 10^5^; Gr4 (most efficient), MLE ≤ 10^6^ RLU/mg protein (Figure 4). For naive mice, the luminescence signal measured was ~10^2^ RLU/mg protein. Nd, not determined; Na, not applicable. See Table S2 for results obtained with other formulations.

## Data Availability

The raw/processed data required to reproduce these findings are either available from supporting material or cannot be shared at this time as the data also form part of ongoing studies.

## Supplementary Materials

The following are available online at https://www.mdpi.com/article/10.3390/pharmaceutics14010025/s1. This includes: (1) Supplementary Experimental Section: in vitro cell cultures; in vitro transfection assays; luciferase and cell viability assays; transaminase assays; CFTR western blot; antibacterial assays. (2) Supplementary Results: synthesis of CholAs; synthesis of CholIm. (3) Supplementary Figures and Tables: Figure S1: Aerosol experimental set-up; Figure S2: DNA condensation and size/zeta measurements before and then after nebulization; Figure S3: Relationships between features of formulations and their gene transfer capabilities; Figure S4: Bioluminescence imaging performed up to 390 days after treatment of mice with a single aerosol of ((CholP/DOPE 1/2 DP5K) + pGM144] CR2 PR2; Figure S5: H&E stain of lung sections after treatment with a single aerosol of [(CholP/DOPE 1/2 DP5K) + pGM144) CR2 PR2; Figure S6: CFTR expression in human bronchial epithelial cell lines and in murine lungs following aerosol treatment with ((CholP/DOPE 1/2 DP5K) + pCIK-CFTR) CR2 PR2; Figure S7: In vitro transfection activity following deposition of lipoplexes mixed with increasing amounts of CF mucus components; Figure S8: Minimal inhibitory concentrations of two aminoglycosides and their cholesterol derivatives towards *S. aureus* and *P. aeruginosa*; Figure S9: In vitro transfection activity following deposition of increasing doses of lipoplexes in the culture medium of bronchial epithelial cell lines; Table S1: Physicochemical characterizations of additional liposomes performed; Table S2: Physico-chemical characterizations of additional lipoplexes performed; Table S3: Physicochemical follow-up during long-term storage at 4 °C of different preparations of two lipidic formulations.
