# Supplementary material for: Aerosol-Mediated Non-Viral Lung Gene Therapy: The Potential of Aminoglycoside-Based Cationic Liposomes
Source: Pharmaceutics. 2021 Dec 23;14(1):25. doi: 10.3390/pharmaceutics14010025 (PMC8778791; doi:10.3390/pharmaceutics14010025)
Supplement: Supplementary file 1 [file pharmaceutics-14-00025-s001.zip › pharmaceutics-1409674-supplementary.pdf]

pharmaceutics

Supplementary Materials: Aerosol-Mediated Non-Viral Lung Gene Therapy: The Potential of Aminoglycoside-Based Cationic Liposomes

Table of contents

1. Supplementary experimental section 2
1.1. In vitro cell cultures 2
1.2. In vitro transfection assays 2
1.3. Luciferase and cell viability assays 2
1.4. CFTR western blot 2
1.5. Transminase assays 2
1.6. In vitro antibacterial assays 2
2. Supplementary Results 3
2.1. Synopsis 3
2.2. Syntheses 3
2.3.1. Synthesis of CholAs 3
2.3.2. Synthesis of CholIm 4
3. Supplementary Figures and Tables 7
4. Supplementary discussion 20
5. References 21
6. List of abbreviations 22

1. Supplementary experimental section

1.1. In vitro cell cultures

Three human epithelial cell lines were used: A549 (ATCC CCL-185), which are adenocarcinoma human alveolar basal epithelial cells; the human bronchial epithelial cells 16HBE-14o^{-}; and the cystic fibrosis bronchial epithelial cells CFBE-41o^{-} [1]. They were grown in DMEM (A549) or EMEM (16HBE and CFBE), supplemented with 10% fetal bovine serum, 1% antibiotic, and 1% L-glutamine (Lonza, Levallois-Perret, France). Incubations were carried out in an incubator at 37 °C in a humidified atmosphere containing 5% CO_2. Mucine isolated from porcine stomach, bovine serum albumin, and low-molecular weight DNA from salmon sperm (Sigma, Saint-Quentin-Fallavier, France) were used to prepare 10 mg/mL stock solutions.

1.2. In vitro transfection assays

Twenty four hours before transfection, cells were seeded in 96 wellplates. Unless otherwise stated, lipoplexes were tested in the form of lipidic formulations as tested in animals. They were deposited dropwise into wells containing cells cultured under submerged condition. A further incubation for 24-48h was done before subsequent assays as described below.

1.3. Luciferase and cell viability assays

Following *in vitro* transfection with the luciferase-encoding pDNA (pGM144), the culture medium was removed and cells were lysed with Passive Lysis Buffer (Promega, Charbonnières-les-Bains, France). Luminescence and total protein content were quantified using the Luciferase Assay System kit (Promega) and the BC assay kit (Interchim, Montluçon, France), respectively. Luciferase expression was expressed as relative light units (RLU) per milligram of total proteins. Cell viability was determined using the Vialight Cell Proliferation & Cytotoxicity BioAssay Kit (Lonza). Results correspond to means +/- SD, with n=3.

1.4. CFTR western blot

Following *in vitro* transfection with the CFTR-encoding pDNA (pCIK-CFTR), the culture medium was removed and cells were lysed and scraped with RIPA solution complemented with Complete Protease Inhibitor cocktail (Roche Diagnostics, Rotkreuz ZG, Switzerland). After dosage of total proteins following Lowry's methodology, 150 μg were separated by SDS-PAGE (7.5%) and transferred to PVDF membranes (GE Healthcare, Tremblay-en-France, France). After blocking with a mixture of milk and Tris-Buffer Sulfate 1X/Tween 0.1%, the membranes were incubated first with either mouse monoclonal anti-CFTR antibody, clone M3A7 (1:500; Merck Millipore, Molsheim, France) or mouse monoclonal anti-Na+/K+ ATPase (α subunit) antibody, clone M7-PB-E9 (1:5000; Merck Millipore) then with HRP-conjugated secondary antibodies (1:20000; Santa-Cruz Biotechnologies, Heidelberg, Germany). A chemiluminescence reaction was performed with Illumina Forte Western HRP Substrate (Merck Millipore) and proteins of interest were visualized with the Chemi-Capt 5000 software (Vilber, Marne-la-Vallée, France).

1.5. Transminase assays

Before sacrificing, some animals were anesthetized by an intraperitoneal injection of ketamine/xylazine (100 and 10 mg/kg body weight, respectively). Whole blood was collected *via* intracardiac puncture. Serum was obtained following overnight storage at 4 °C followed by centrifugation to collect the supernatant. Transaminase (alanine transaminase, ALT, and aspartate transaminase, AST) dosages were performed using the Elitech kit together with the Selectra E apparatus (Elitech, Puteaux, France), according to the manufacturer's instructions and as previously reported [2,3]. Results were expressed as International Units per Liter (IU/L).

1.6. In vitro antibacterial assays

Staphylococcus aureus and *Pseudomonas aeruginosa* bacterial strains were used: RN4220 (ATCC 35556) [4] and Newman (ATCC 25904) [5] are laboratory and clinical *S. aureus* strains, respectively; PAL is a clinical *P. aeruginosa* strain that we have isolated from a CF patient sputum [6]. Minimal inhibitory concentrations (MIC) of lipidic formulations or aminoglycosides were determined using a liquid broth micro-dilution method, as previously reported [6].

2. Supplementary Results

2.1. Synopsis

This study was conducted following the experimental plan depicted below, which allowed to highlight the potential of aminoglycoside-based cationic liposomes for aerosol mediated lung gene therapy.

Experimental plan	Main results obtained
First studies using a panel of diverse lipids	
1) Selection of 11 cationic lipids and 5 colipids	
	⇒ Report of 2 original cationic lipids
2) Preparation of a series of lipidic formulations at high concentration	
	⇒ Selection of highly concentrated stable formulations
3) Mixture of stable lipidic formulations with DNA at high concentration	
	⇒ Selection of highly concentrated stable lipid/DNA mixtures
4) Aerosol delivery in mice lungs for safety and efficacy assessments	
	⇒ Safety for every aerosol
	⇒ Demonstration of diverse abilities to transfect mice lungs
	⇒ Identification of CholP/DOPE as a lead formulation
Further studies focusing on CholP/DOPE formulation	
• *In vivo*: longitudinal follow-up following a single aerosol treatment	
	⇒ Sustained luciferase transgene expression in mice lungs
• *In vivo*: histology for efficacy and safety assessments following a single aerosol treatment	
	⇒ Widespread safe transgene expression in lungs
• *In vitro* and *in vivo*: aerosol delivery of a CFTR-encoding pDNA	
	⇒ Strong CFTR expression in CFBE cells (*in vitro*)
	⇒ Discrete CFTR expression in mice lungs (*in vivo*)
• *In vitro*: evaluations under conditions closer to CF airways	
	⇒ Moderate resistance to mucus components
• *In vitro*: antibacterial assays using clinical bacterial strains	
	⇒ No impact on antibiotic resistance in *S. aureus* and *P. aeruginosa*

Synopsis. Rational experimental plan and main results obtained.

2.2. Syntheses

The cholesterol-based trimethylarsonium cationic lipid CholAs and the cholesterol-based imidazolium cationic lipid CholIm were obtained following two-step synthetic routes, as illustrated in Scheme 1 (see manuscript paper) and further detailed below.

***N*-(2-iodoethyl) carbamoyl cholesterol (Step 1).** Cholesteryl chloroformate (2.0 g, 4.50 mmol) and 2-iodoethanol (2.18 g, 10.60 mmol) were dissolved in dry DCM (20 mL) at 0 °C (acetone/ice). Following addition of pyridine (1 mL), the reaction was stirred for 1 h at 0 °C and then overnight at RT. The organic solution was washed three times with 1 N HCl (2 × 30 mL), water (2 × 30 mL), dried with $MgSO_4$, then filtered and concentrated. The crude was recrystallized from DCM/methanol to give a white powder (2.40 g, 92%). ^{1}H NMR (400 MHz, $CDCl_3$): δ 0.65 (s, 3H), 0.83-1.53 (m, 32H), 1.84-1.97 (m, 5H), 2.40 (m, 2H), 3.30 (t, 2H, *J*= 7.0 Hz), 4.37 (t, 2H, *J*= 7.0 Hz), 4.49 (m, 1H), 4.57 (m, 2H), 5.39 (d, 1H) [7].

2.3.1. Synthesis of CholAs

***N*-(2-trimethylarsonium ethyl) carbamoyl cholesterol (CholAs; Step 2a).** *N*-(2-iodoethyl) carbamoyl cholesterol (500 mg, 0.86 mmol) was dissolved in dry THF (2 mL) and trimethylarsine (207 mg, 1.72 mmol) was added. The reaction was refluxed for 72 h at 60 °C. Solvent was evaporated and the residue was purified by flash column chromatography with 0-10% methanol in DCM to afford a white powder (362 mg, 60%). ^{1}H NMR (400 MHz, $CDCl_3$): δ 0.65 (s, 3H), 0.83-1.53 (m, 32H), 1.84-1.97 (m, 5H), 2.29 (s, 9H), 2.34 (m, 2H), 3.26 (t, 2H, *J*= 6.0 Hz), 4.50 (m, 1H), 4.57 (m, 2H), 5.36 (d, 1H). ^{13}C NMR (162 MHz, $CDCl_3$): 10.1, 11.7, 18.6, 19.1, 20.9, 22.4, 22.7, 23.7, 24.1, 26.4, 27.5, 27.9, 28.1, 31.7, 31.8, 35.7, 36.0, 36.4, 36.6, 37.8, 39.4, 39.6, 42.2, 49.8, 56.0, 56.5, 61.7, 78.8, 123.2, 138.8, 153.3. HRMS (Maldi-TOF) *m/z*: calc. For $C_{33}H_{58}AsIO_3$: 704.265 $[M+H]^+$ Found : 704.638 (Spectra 1).

2.3.2. Synthesis of CholIm

***N*-(1-methylimidazolium ethyl) carbamoyl cholesterol (CholIm; Step 2b).** *N*-(2-iodoethyl) carbamoyl cholesterol (1.0 g, 1.71 mmol) was suspended in an excess of *N*-methylimidazole (5 mL) and the reaction was stirred at 40 °C for 20 h. The end of the reaction was assessed by TLC (DCM/MeOH 10%). The excess of *N*-methylimidazole was evaporated by bubble-to-bubble distillation (100 °C, 7.2 10^{-2} Torr, 1 h) and the residue was purified by flash column chromatography with DCM/MeOH 0-10% to afford a brown waxy solid (684 mg, 60%). ^{1}H NMR (400 MHz, $CDCl_3$): δ 0.66 (s, 3H), 0.83-1.73 (m, 46H), 2.35 (s, 2H), 4.13 (s, 3H), 4.30 (m, 1H), 4.52 (t, 2H, *J*= 7.0 Hz), 4.87 (t, 2H, *J*= 7.0 Hz), 5.39 (s, 1H), 7.34 (s, 1H), 7.52 (s, 1H), 10.01 (s, 1H). ^{13}C NMR (162 MHz, $CDCl_3$): 11.4, 18.2, 18.8, 22.1, 22.3, 27.5, 27.8, 35.3, 36.0, 36.9, 37.4, 39.0, 41.8, 48.6, 49.5, 55.7, 56.2, 64.9, 78.3, 122.7, 123.5, 138.6, 153.1 (Spectra 2).

A)

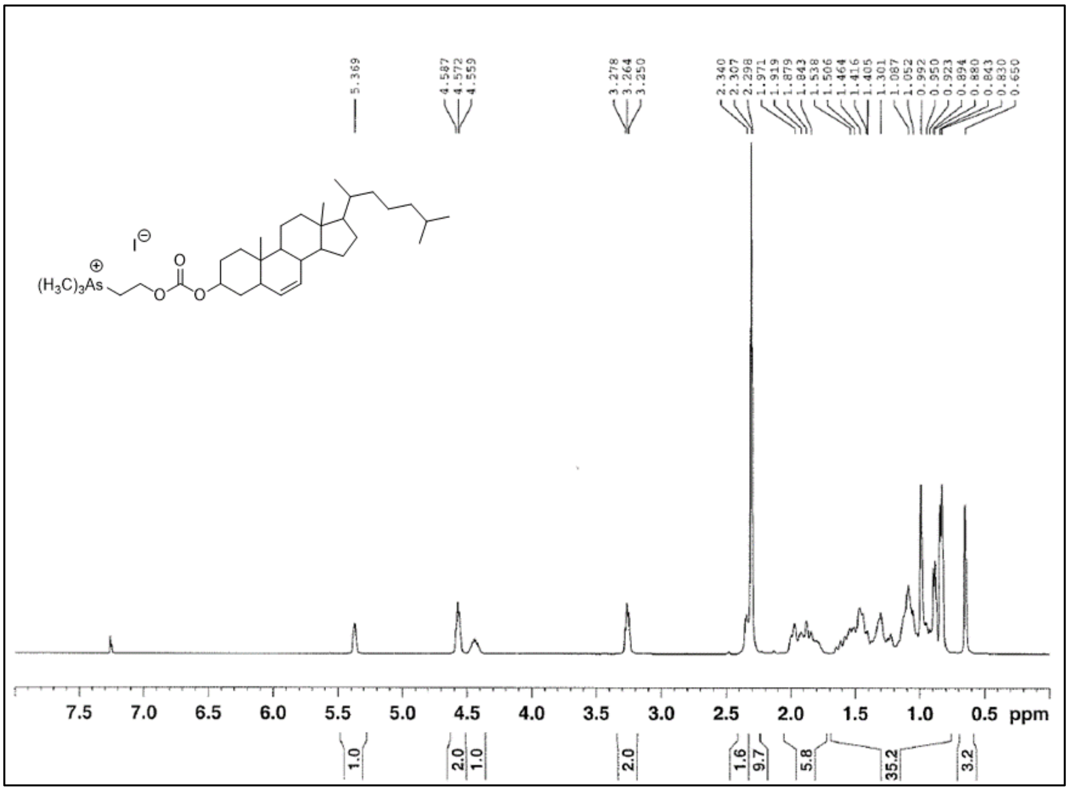

B)

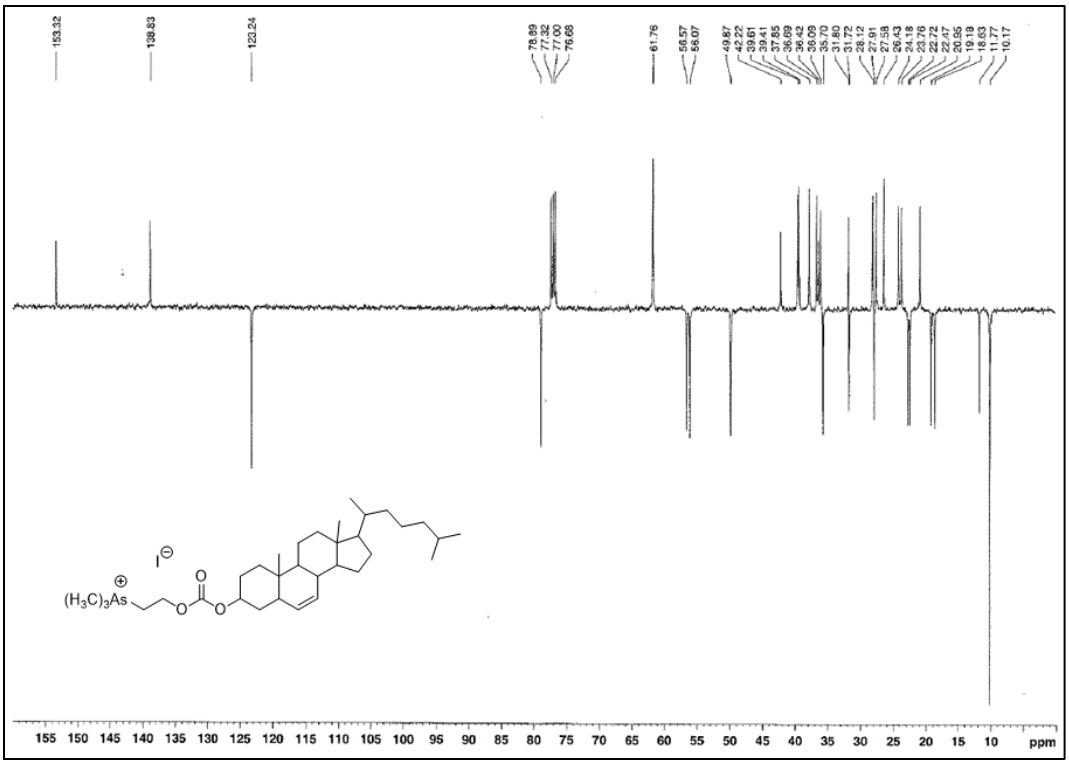

Spectra 1. ^{1}H NMR (A) and ^{13}C NMR (B) spectra of CholAs.

A)

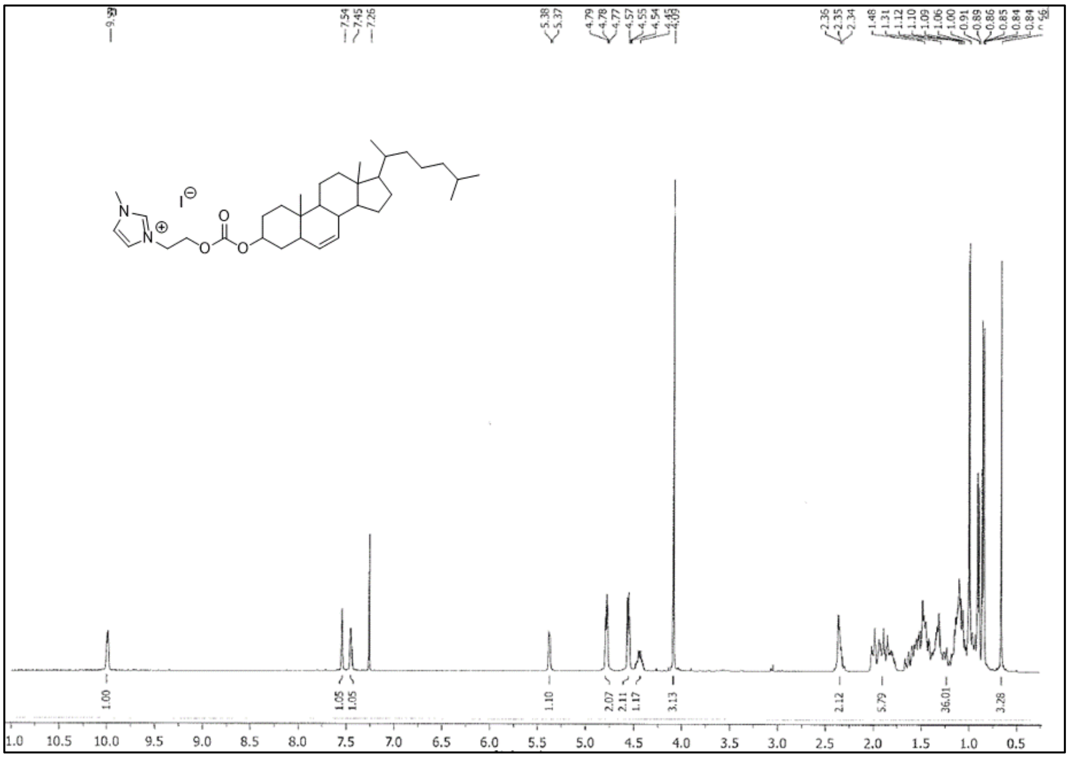

B)

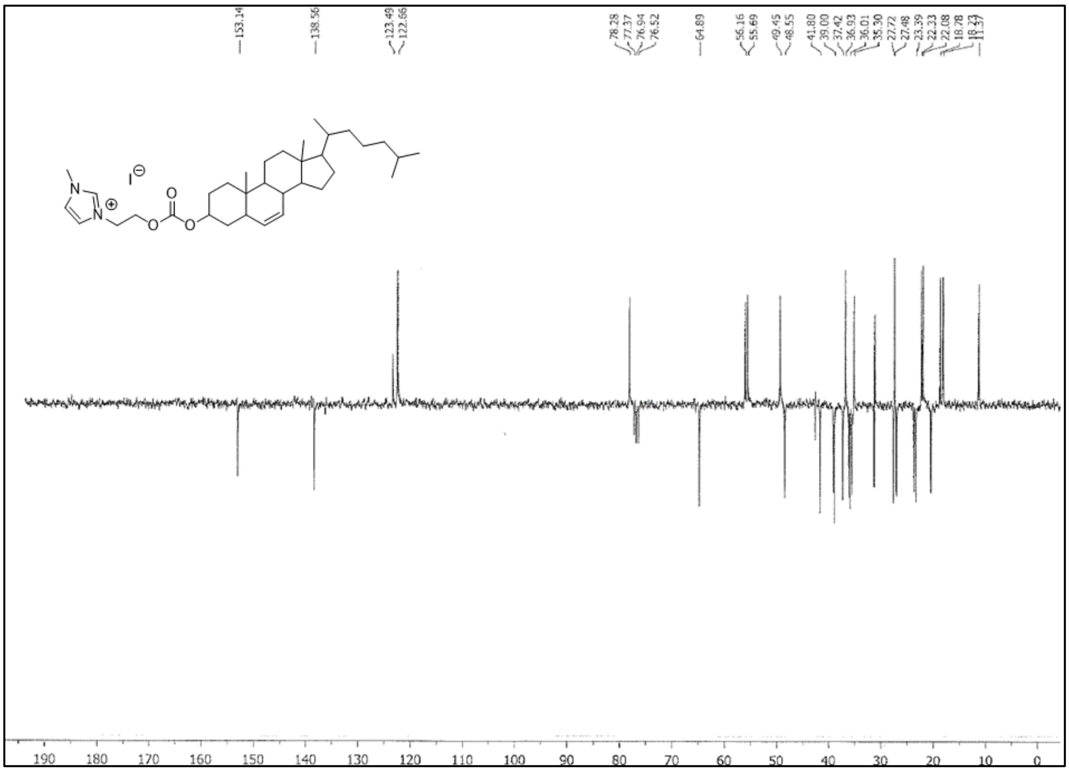

Spectra 2. ^{1}H NMR (A) and ^{13}C NMR (B) spectra of CholIm.

3. Supplementary Figures and Tables

Figure S1. Aerosol experimental set-up.

Figure S2. DNA condensation (panel A) and size/zeta measurements (Table B) before (1) and then after (2) nebulization.

Figure S3. Relationships between features of formulations and their gene transfer capabilities.

Figure S4. Bioluminescence imaging performed up to 390 days after treatment of mice with a single aerosol of [(CholP/DOPE 1/2 DP5K)+pGM144] CR2 PR2.

Figure S5. H&E stain of trachea and lung sections.

Figure S6. CFTR expression in human bronchial epithelial cell lines and in murine lungs following aerosol treatment with [(CholP/DOPE 1/2 DP5K) + pCIK-CFTR] CR2 PR2.

Figure S7. *In vitro* transfection activity following deposition of lipoplexes mixed with increasing amounts of CF mucus components.

Figure S8. Minimal inhibitory concentrations of two aminoglycosides and their cholesterol derivatives towards *S. aureus* and *P. aeruginosa*.

Figure S9. *In vitro* transfection activity following deposition of increasing doses of lipoplexes in the culture medium of bronchial epithelial cell lines.

Table S1. Composition and physicochemical characterizations of additional liposomes performed (but not tested in animals).

Table S2. Composition and physicochemical characterizations of additional lipoplexes performed (but not tested in animals).

Table S3. Physicochemical follow-up during long-term storage at 4 °C of different preparations of two lipidic formulations (without DNA).

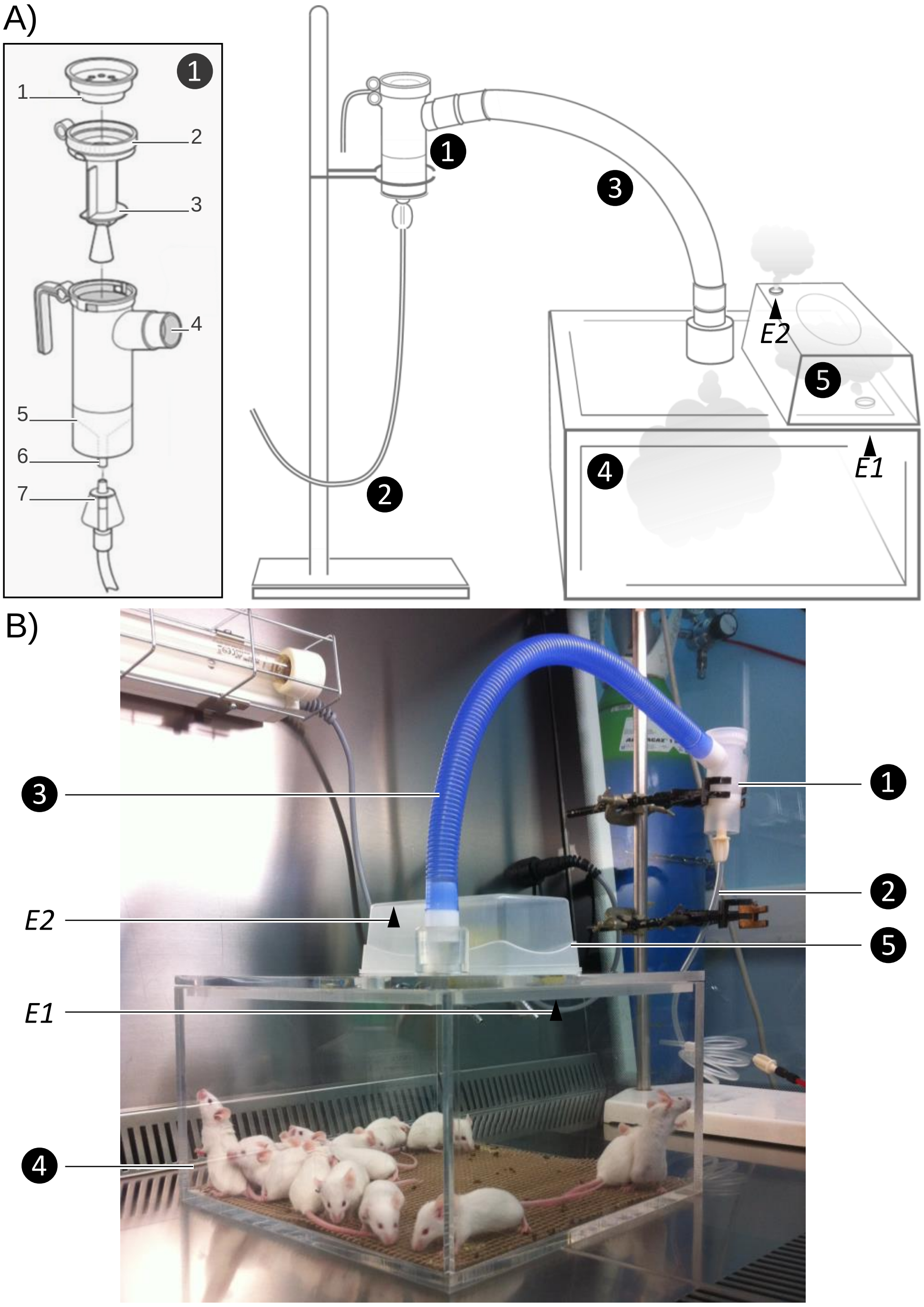

Figure S1. Aerosol experimental set-up. The schematic illustration in panel A shows the clinically-validated PARI LC PLUS nebulizer (https://www.pari.com/int/products/nebuliser-and-year-packs/pari-lc-plus-nebuliser/) ❶ (comprising: 1, inspiratory valve cap; 2, insert; 3, semi-circle; 4, outlet; 5, reservoir; 6, air intake; 7, wing tip tubing) connected on one side to a flexible hose (supplying 2 bars pressurized air) ❷ and on the other side to a 55 cm tube ❸ conducting the aerosol generated to a Perspex box ❹ where mice can be housed. The aerosol reaches *via* an exit E1 a second compartment ❺ fitted over the cover of the exposure box. This second compartment allows exposure of other biological materials and to collect a sample of the aerosol. The aerosol finally goes out of the system *via* an exit E2. The picture in B shows the set-up placed under a hood, during aerosol treatment of 15 BALB/c mice.

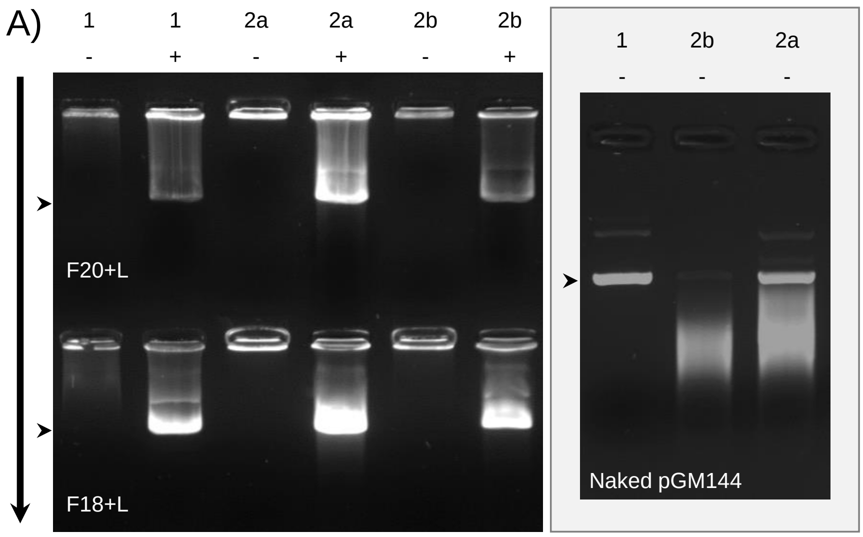

B)

Formulation	Size (nm)		PDI		Zeta (mV)	
	Mean	SD	Mean	SD	Mean	SD
F20	82	1	0.21	0.01	+37	1
F20+L (1 -)	89	1	0.22	0.01	+15	1
F20+L (2a -)	122	1	0.20	0.01	+10	1
F20+L (2b -)	102	2	0.23	0.01	+15	1

Formulation	Size (nm)		PDI		Zeta (mV)	
	Mean	SD	Mean	SD	Mean	SD
F18	294	3	0.27	0.02	+55	1
F18+L (1 -)	262	5	0.22	0.04	+30	1
F18+L (2a -)	265	3	0.27	0.01	+26	1
F18+L (2b -)	332	7	0.33	0.08	+26	1

Figure S2. Gel retardation (panel A) and size/zeta measurements (Table B) before (1) and then after (2) nebulization: representative results obtained with [(CholP/DOPE 1/2 DP5K) + pGM144] CR2 PR2 (F20+L) and [(GL67/DOPE 1/2 DP5K) + pGM144] CR2 PR1 (F18+L). The samples of lipoplexes assayed after nebulization correspond to either an aliquot of the volume remaining in the nebulizer reservoir (2a) or an aliquot of the aerosol collected in the exposure box (2b). In panel A, samples were assayed following incubation with (+) dextran sulfate or (-) water; as control, naked pDNA was evaluated in parallel to show its degradation during nebulization. A vertical arrow denotes the direction of the electrical field. An arrowhead indicates the position of the supercoiled pDNA form in the gel. Fluorescence observed above or below that form in a given lane correspond to pDNA either partly relaxed or degraded, respectively. In Table B, measurements are also provided for liposomes of each formulation (without pDNA).

A) Characteristics of lipid+pDNA formulations

#	CL	Z+a	LDb	LogPc	Colipid(s)	MRd	PRe	CRf
F1+L	CholAs	Pc(1)	Chol	9.2	None	Na	1	1
F2+L	CholIm	Pc(1)	Chol	4.5	DOPE	3/2	2	2
F3+L	DOSP	Pa(4)	Oleic	-6.8	DOPI	1/1	1	1
F4+L	DOPIm	Pc(1)	Oleic	10.1	DOPE/Chol	1/1/1	2	2
F5+L	DOPAs	Pc(1)	Oleic	14.2	None	Na	1	2
F6+L	DOSP	Pa(4)	Oleic	-6.8	DOPE	1/1	2	2
F7+L	DOSP	Pa(4)	Oleic	-6.8	Tetraether	5/1	2	2
F8+L	DOGB	Pc(1)	Oleic	8.5	Tetraether	10/1	1	2
F9+L	DOSP	Pa(4)	Oleic	-6.8	DOPI	1/1	2	4
F10+L	DOSP	Pa(4)	Oleic	-6.8	DOPI	1/1	1	2
F11+L	CholP	Pa(4)	Chol	-12.4	Diether	1/2	2	2
F12+L	DOPIm	Pc(1)	Oleic	10.1	DOPI	1/1	1	2
F13+L	CholRi	Pa(3)	Chol	-7.5	DOPE	1/2	2	2
F14+L	CholKB	Pa(4)	Chol	-11.2	DOPE	1/2	2	2
F15+L	CholP	Pa(4)	Chol	-12.4	DOPE	1/1	2	2
F16+L	CholP	Pa(4)	Chol	-12.4	DOPI	1/1	2	2
F17+L	CholK	Pa(3)	Chol	-8.1	DOPE	1/2	2	2
F18+L	GL67	Pa(2)	Chol	0.9	DOPE	1/2	1	2
F19+L	CholT	Pa(4)	Chol	-10.5	DOPE	1/2	2	2
F20+L	CholP	Pa(4)	Chol	-12.4	DOPE	1/2	2	2

a, Protonatable amine (Pa) or permanent cation (Pc) in cationic lipid headgroup (number of positive charge at physiological pH 7.4); b, Lipid domain of cationic lipid; c, Partitioning value of cationic lipid ionic species at physiological pH 7.4 (MarvinSketch 5.11.5, Chemaxon, http://www.chemaxon.com); d, Cationic lipid/colipid(s) mass ratio; e, DP5K/DNA mass ratio; f, Cationic lipid/pDNA charge ratio.

B) Luciferase activity in lung homogenates

Each square corresponds to one animal

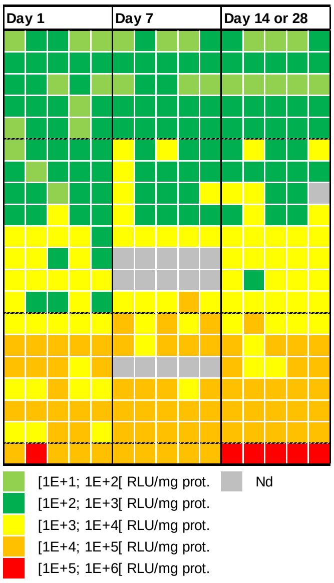

C) Ratio of maximal luciferase expressions (MLE)

Considering the mean of expressions on day 1, 7, and 14/28

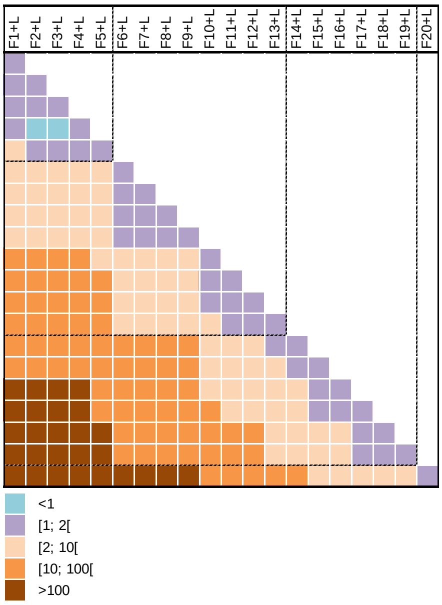

D) Pairwise comparisons for highlighting the individual effect of formulation features on gene delivery efficiency in murine lungs

Features compared	Formulations compared	Day 1 Ratio	Day 1 *p*-value	Day 7 Ratio	Day 7 *p*-value	Day14/28 Ratio	Day14/28 *p*-value
a) CL with protonatable amines *versus* CL with a permanent cation	All / all[1]	11	<0.0001	49	<0.0001	38	<0.0001
b) CL with a cholesterol moiety *versus* CL with two oleic chains	F5+L (DOPAs) / F1+L (CholAs)	1	0.8016	3	0.0952	1	0.5317
	F12+L (DOPIm) / F2+L (CholIm)	30	0.0079	Na	Na	10	0.0952
	F16+L (CholP) / F10+L (DOSP)	10	0.0079	Na	Na	5	0.0079
	F20+L (CholP) / F6+L (DOSP)	403	0.0079	112	0.0079	171	0.0079
c) CL with LogP <1 *versus* CL with LogP >1	All / all[2]	10	<0.0001	40	<0.0001	34	<0.0001
d) CholP/DOPE 1/2 *versus* GL67A	F20+L (CholP) / F18+L (GL67)	5	0.0079	2	0.0079	3	0.0079
e) Charge ratio of lipid/DNA mixture	F10+L (CR2) / F9+L (CR4)	5	0.0159	2	0.0952	3	0.0317
	F10+L (CR2) / F3+L (CR1)	10	0.0079	22	0.0079	68	0.0079
f) CL/DOPE molar ratio 1/1 *versus* 1/2	F20+L (1/2) / F15+L (1/1)	4	0.0079	6	0.0079	11	0.0079
g) Colipid combined to the CL	F20+L (DOPE) / F11+L (Diether)	43	0.0079	Na	Na	32	0.0079
	F20+L (DOPE) / F16+L (DOPI)	3	0.0079	Na	Na	10	0.0079
	F10+L (DOPI) / F6+L (DOPE)	14	0.0079	3	0.0317	4	0.0317
	F10+L (DOPE) / F7+L (Tetraether)	8	0.0079	2	0.0317	6	0.0079
h) Aminoglycoside headgroup in CL	F20+L (Paromomycin) / F19+L (Tobramycin)	9	0.0079	3	0.0079	3	0.0079
	F19+L (Tobramycin) / F17+L (Kanamycin)	1	0.5317	1	0.6667	2	0.3095
	F17+L (Kanamycin) / F14+L (Kanamyine B)	2	0.8016	2	0.2222	5	0.0079
	F14+L (Kanamycin B) / F13+L (Ribostamycin)	3	0.0556	1	0.3095	1	0.8016

Ratio are calculated considering the mean of luciferase expressions in the two groups compared (max/min). Statistically significant differences (*p*-value<0.05) are shown in red.

1, (F6+L, F7+L, F3+L, F9+L, F10+L, F11+L, F13+L, F14+L, F15+L, F16+L, F17+L, F18+L, F19+L, F20+L) / (F1+L, F2+L, F4+L, F5+L, F8+L, F12+L)

2, (F3+L, F6+L, F7+L, F9+L, F10+L, F11+L, F13+L, F14+L, F15+L, F16+L, F17+L, F19+L, F20+L) / (F1+L, F2+L, F4+L, F5+L, F8+L, F12+L)

Figure S3. Relationships between characteristics and gene transfer capabilities of the formulations evaluated. Panel A collates relevant information characterizing the main formulations assayed in animals. In panel B, luciferase expression measured in lung homogenates are color-coded according to their intensity. In panel C, the mean luciferase expression was determined on day 1, 7, and 14 or 28 for every formulation. The maximum was then used to calculate the ratio value for every possible two comparison. In panel D, pairwise comparisons of luciferase expression highlight the individual contribution of various parameters to the gene transfer efficiency in murine lungs.

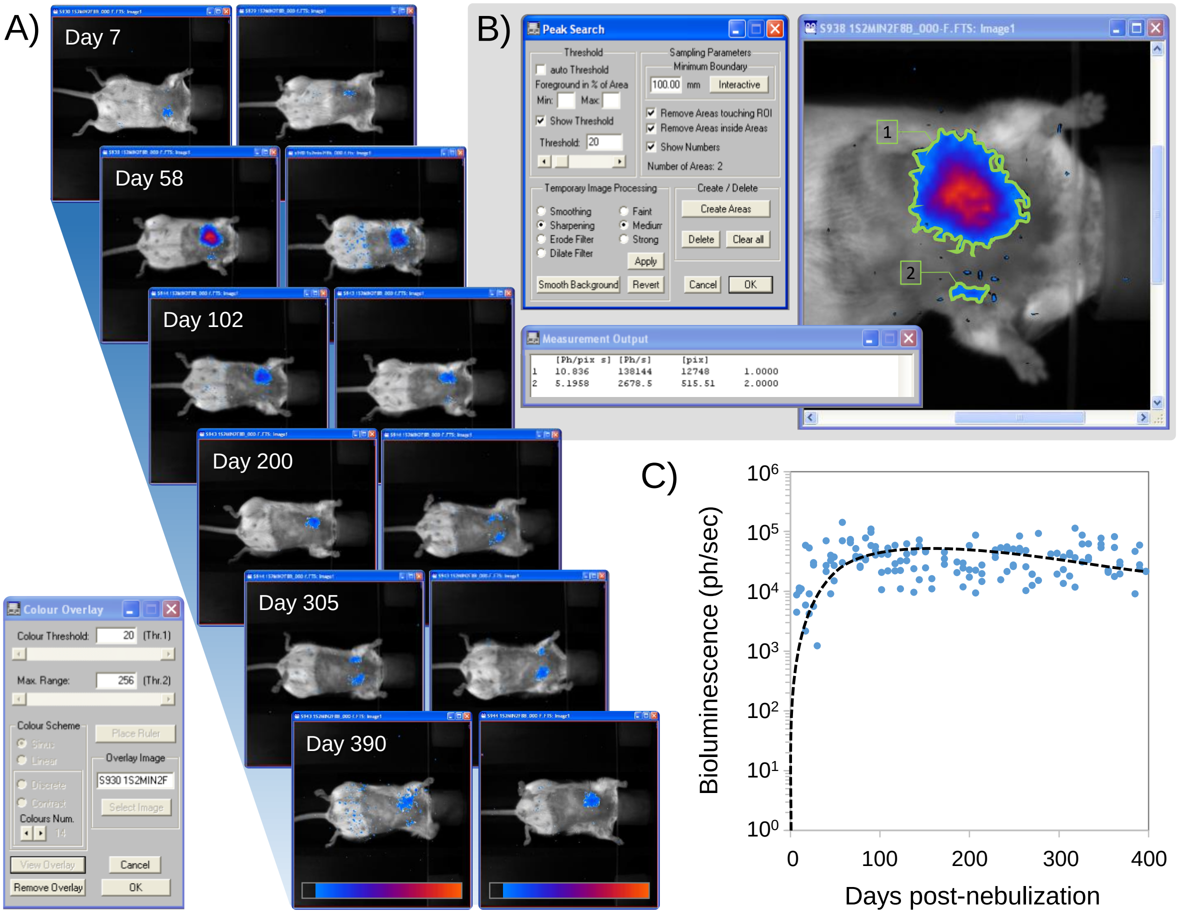

Figure S4. Bioluminescence imaging performed up to 390 days after treatment of mice with a single aerosol of [(CholP/DOPE 1/2 DP5K)+pGM144] CR2 PR2. BLI was done from the 7th day after treatment and then repeated approximately every week. At each time point, three animals were imaged (seventeen in total being considered). Panel A shows representative pictures captured 7, 58, 102, 200, 305, and 390 days post-aerosol. Panel B illustrates the procedure used to quantify luminescence in minimal surface areas. This allowed specific consideration of transgene expression likely originating from the right and/or left lungs. In panel C, luminescence signals are reported over time. Each dot corresponds to a distinct patch of a given animal at one time point after aerosol. The dashed line refers to the trend of luminescence emission in all animals.

Additional comments: At all the time points considered after aerosol treatment, luminescence could be detected only from treated animals, appearing as one or two distinct patches at the surface of the chest area. In most cases, luminescence collected from the right side was stronger than that from the left side of animals; this was likely due to the smaller size of the left lung lobes as well as photon absorption by the heart.

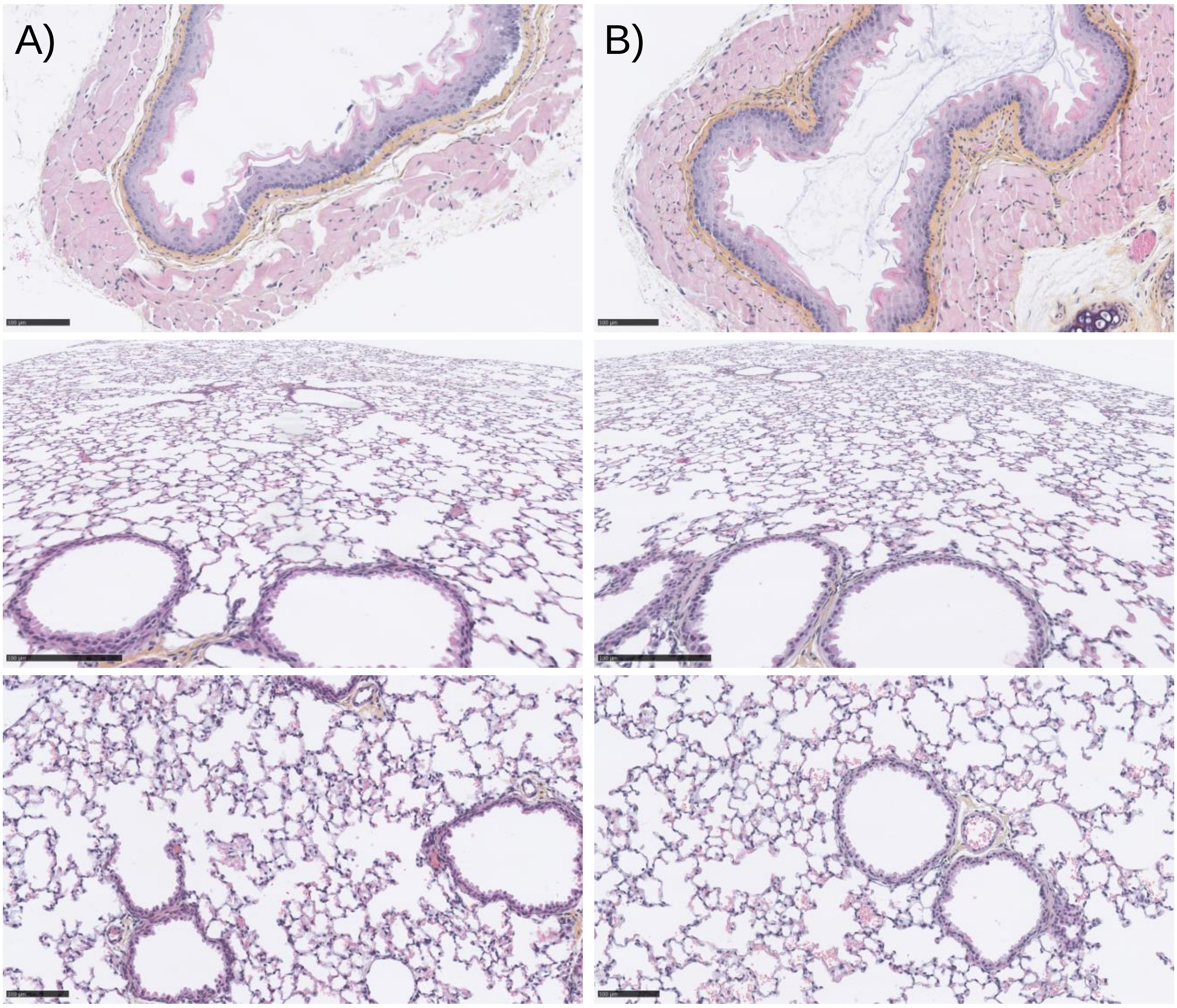

Figure S5. H&E stain of trachea and lung sections. Images correspond to mice treated with a single aerosol of [(CholP/DOPE 1/2 DP5K)+pGM144] CR2 PR2 (A) or untreated mice (B). The scale bar is 100 μm.

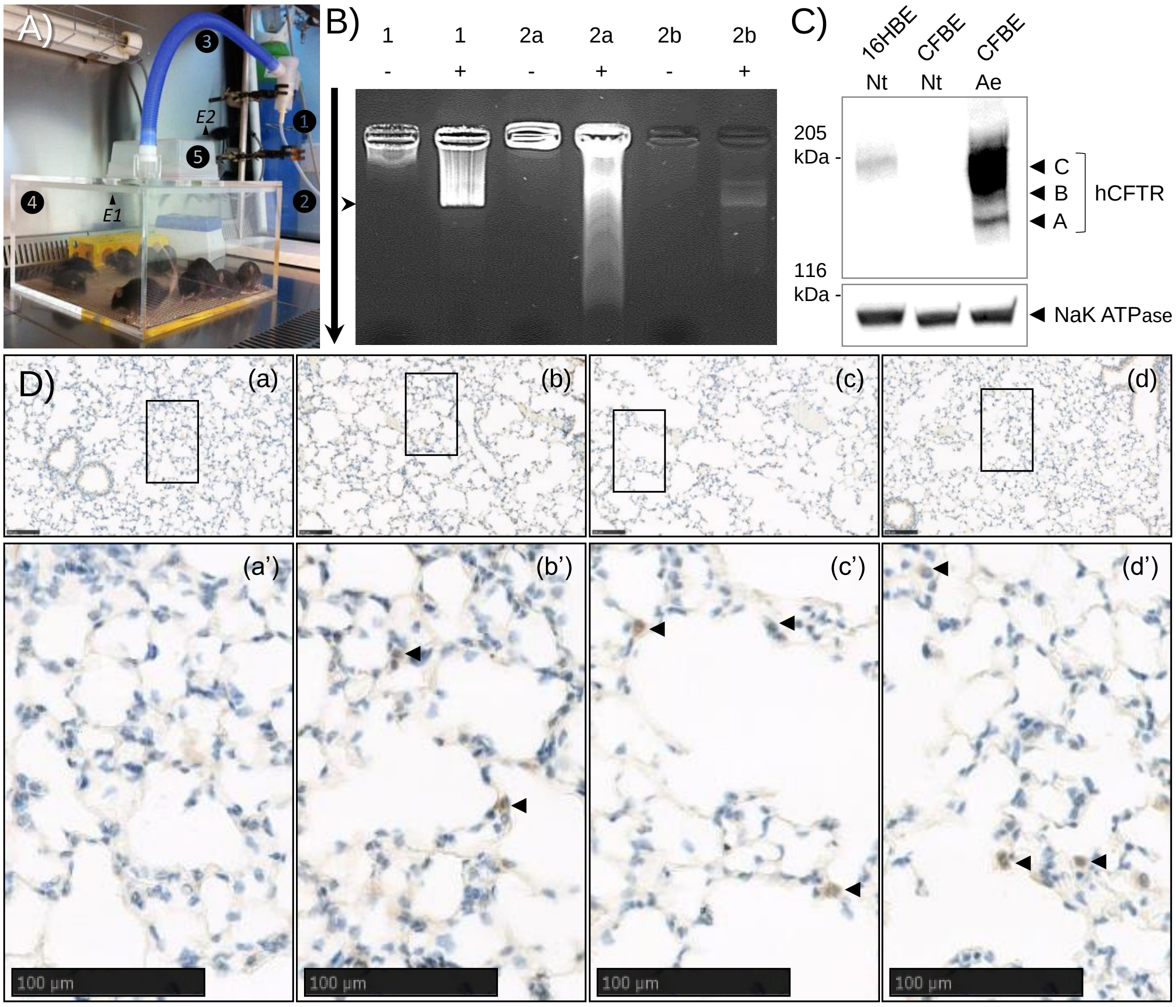

Figure S6. CFTR expression in human bronchial epithelial cell lines and in murine lungs following aerosol treatment with [(CholP/DOPE 1/2 DP5K) + pCIK-CFTR] CR2 PR2. Panel A shows the aerosol experimental set-up used for hosting both *Cftr*$^{-/-}$ mice (in the transparent box) and human bronchial epithelial cells (in the second compartment fitted over the cover of the latter). In panel B, pDNA condensation was assayed in lipoplexes before (1) and after (2) nebulization (in samples collected from the volume remaining in the nebulizer reservoir (2a) or collected in the exposure box (2b)), and following incubation with (+) dextran sulfate or (-) water. A vertical arrow denotes the direction of the electrical field and an arrowhead indicates the position of the supercoiled form of pDNA. Panel C illustrates immunoblot-detection of hCFTR protein in CFBE cells exposed to the aerosol (Ae) or not (Nt); 16HBE cells were used as positive (hCFTR-expressing) control. Panel D provides representative immunohistochemistry pictures showing hCFTR protein detection in untreated (a) or treated (b, c, d) murine lungs. Squared areas in (a), (b), (c), and (d) are magnified in (a'), (b'), (c'), and (d'), respectively. The scale bar is 100 µm in every case. Black arrowheads point to hCFTR-positive cells.

<u>Additional comments:</u> Compared to pGM144, pCIK-CFTR was not as optimized for *in vivo* studies since it notably features numerous CpG dinucleotides. Taking into account this limitation, mice were sacrificed early – i.e. 24 h – after aerosol treatment, in order to limit the effect of some inflammatory response against both pDNA CpG motifs and the transgene delivered [3,8]. Cells positive for the hCFTR deriving from the pCIK-CFTR was specifically detected in the lungs of almost all treated animals. Under these experimental conditions, CFTR expression levels were similar in animals that were treated with either GL67A or CholP-based formulation.

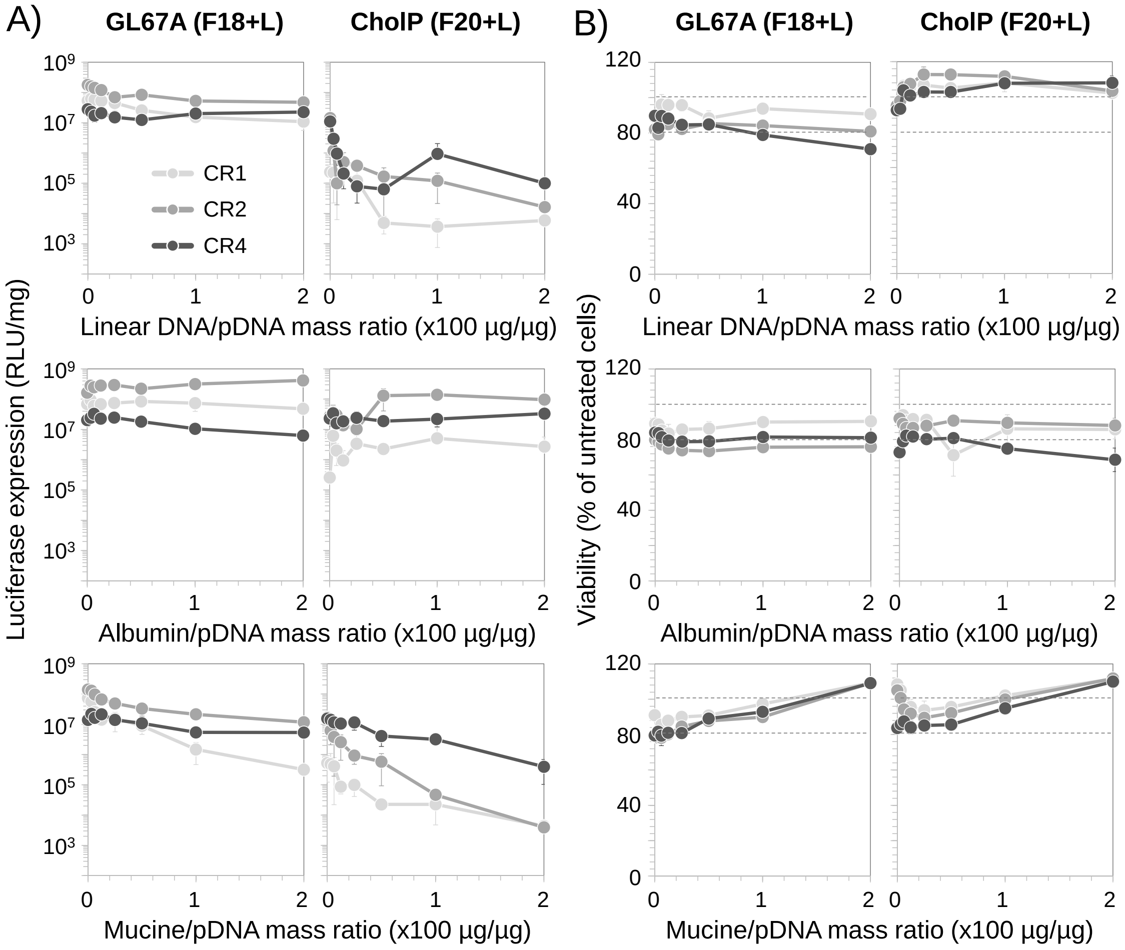

Figure S7. *In vitro* transfection activity following deposition of lipoplexes mixed with increasing amounts of CF mucus components. CholP and GL67A were tested in the form of lipidic formulations as tested in animals, i.e. [(CholP/DOPE 1/2 DP5K) + pGM144] CR2 PR2 (F20+L) and [(GL67/DOPE 1/2 DP5K) + pGM144] CR2 PR1 (F18+L), respectively. In addition, other lipoplexes were formed at CR1 (i.e. [(CholP/DOPE 1/2 DP5K) + pGM144] CR1 PR1 and [(GL67/DOPE 1/2 DP5K) + pGM144] CR1 PR0.5) or CR4 (i.e. [(CholP/DOPE 1/2 DP5K) + pGM144] CR4 PR4 and [(GL67/DOPE 1/2 DP5K) + pGM144] CR4 PR2). In every case, 50 µL incorporating 2 µg of complexed pDNA mixed with a given dose of either linear DNA, albumin or mucine (up to 400 µg per well) were deposited per well previously seeded with CFBE cells. By doing so, CF mucus component to pDNA mass ratios from 0 to 200 were assayed. Transfection activity (i.e. reporter gene expression, A, and cell viability, B) was determined 24 h later. In panel B, the upper and lower dashed lines specify 100 and 75% cell viability, respectively. Results correspond to means +/- SD, with n=3.

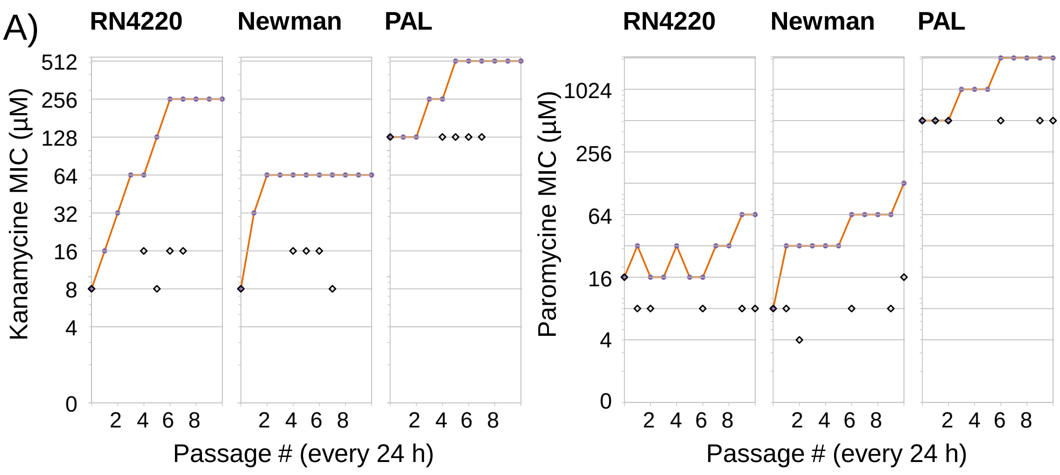

B)

		Repeated exposure to		
		**Water (no drug)**	**CholK (100 µM)**	**Kana (MIC/2)**
RN4220	Kanamycin MIC (µM)	8-16	8-16	256
	CholK MIC (µM)	>1024	>1024	>1024
Newman	Kanamycin MIC (µM)	8-16	8-16	64
	CholK MIC (µM)	>1024	>1024	>1024
PAL	Kanamycin MIC (µM)	128	128	512
	CholK MIC (µM)	>1024	>1024	>1024

C)

		Repeated exposure to		
		**Water (no drug)**	**CholP (100 µM)**	**Paro (MIC/2)**
RN4220	Paromomycin MIC (µM)	8-16	8-16	64
	CholP MIC (µM)	>1024	>1024	>1024
Newman	Paromomycin MIC (µM)	4-16	4-16	128
	CholP MIC (CholP)	>1024	>1024	>1024
PAL	Paromomycin MIC (µM)	512	512	2048
	CholP MIC (CholP)	>1024	>1024	>1024

Figure S8. Minimal inhibitory concentrations of two aminoglycosides and their cholesterol derivatives towards *S. aureus* and *P. aeruginosa*. RN4220 [4] and Newman [5] are laboratory and clinical *S. aureus* strains, respectively. PAL is a clinical *P. aeruginosa* strain isolated from a CF patient sputum [6]. CholP and CholK were tested in the form of lipidic formulations as tested in animals, i.e. CholP/DOPE 1/2 DP5K (F20) and CholK/DOPE 1/2 DP5K (F17), respectively. Panel A shows the evolution of susceptibility during ten successive passages of bacteria to subinhibitory concentrations of either Kanamycin (left) or Paromomycin (right). MIC were determined every day (red lines), considering concentrations from 4 to 2048 µM. As controls (single black dots), MIC of wild type (i.e. naïve non-exposed to Kanamycin or Paromomycin) bacteria were checked at several time points. Panels B and C provide MIC values determined after repeated incubations (during five successive passages) of bacteria to either water, CholP (100 µM), CholK (100 µM), or sub-inhibitory concentrations (MIC/2) of Kanamycin or Paromomycin. MIC, minimal inhibitory concentration; Nd, not determined.

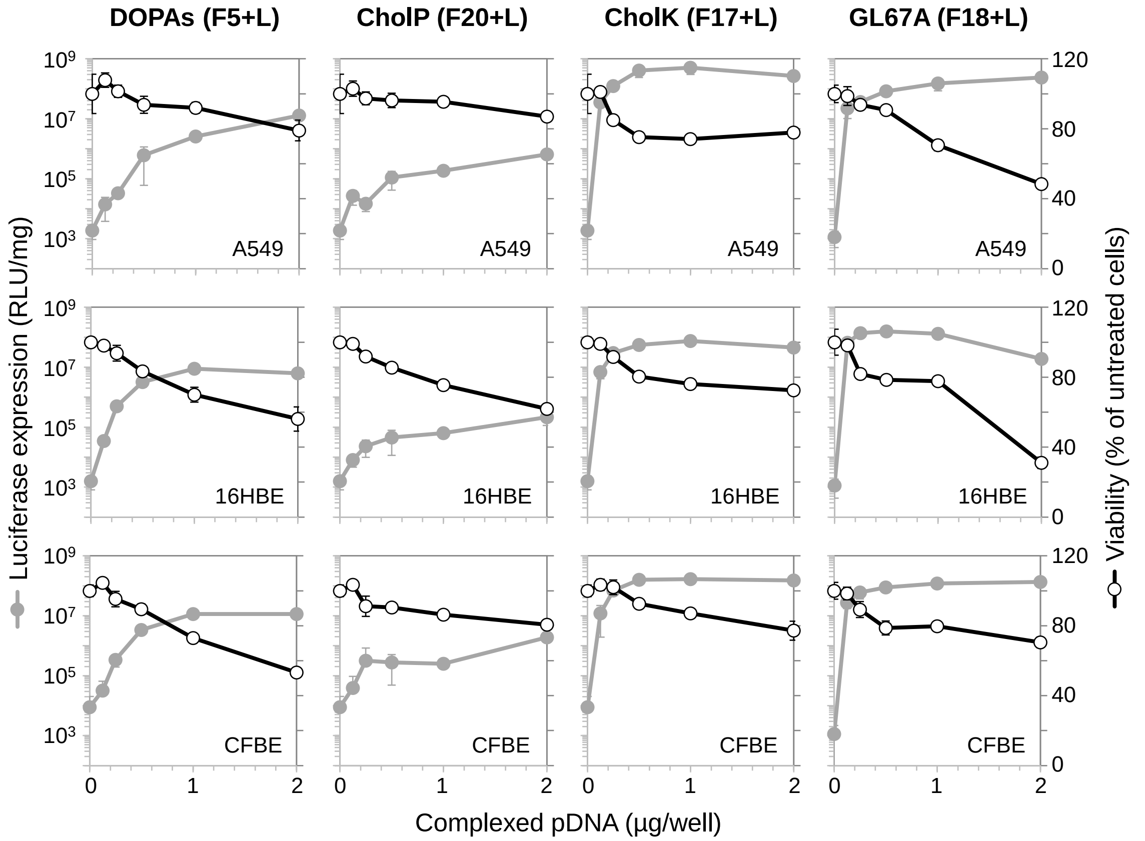

Figure S9. *In vitro* transfection activity following deposition of increasing doses of lipoplexes in the culture medium of bronchial epithelial cell lines. DOPAs, CholP, CholK and GL67A were tested in the form of lipidic formulations as tested in animals, i.e. [(DOPAs DP5K) + pGM144] CR2 PR1 (F5+L), [(CholP/DOPE 1/2 DP5K) + pGM144] CR2 PR2 (F20+L), [(CholK/DOPE 1/2 DP5K) + pGM144] CR2 PR2 (F17+L), and [(GL67/DOPE 1/2 DP5K) + pGM144] CR2 PR1 (F18+L), respectively. Increasing amounts of lipoplexes (corresponding to 0.125, 0.25, 0.5, 1 or 2 µg of complexed pDNA) were added dropwise into the culture medium of A549, 16HBE, and CFBE. Transfection activity (i.e. reporter gene expression and cell viability) was determined 24 h later. Results correspond to means +/- SD, with n=3.

Additional comments: Although CholP-based formulation (F20+L) is the most efficient to perform aerosol gene transfer in lungs (**Figure 4**), it is clearly not the best system for DNA transfection in cell lines (**Figure S9**). This finding is in accordance with results obtained in another *in vitro* study that compared cholesterol paromomycin with other aminoglycoside derived cationic lipids [9]. By contrast, DOPAs-based formulation (F5+L) is quite efficient for cell transfection *in vitro* (**Figure S9**) but ineffective for lung transfection *via* aerosol (**Figure 4**). Altogether, these results clearly show the discrepancy that can be found between *in vitro* and *in vivo* conditions. It emphasizes the importance of experimental conditions that approach as closely as possible the clinical settings to assess the potential of a gene delivery system for therapeutic application.

Table S1. Composition and physicochemical characterizations of additional liposomes performed (but not tested in animals).

Lipidic formulation composition					Physicochemistry		
CL	[CL][a]	Colipid(s)[b]	MR[c]	[DP5K][d]	Size[e]	PdI[f]	Zeta[g]
DOPAs	30	DOPE	1/1	5	217	0.33	+59
CholAs	15	DOPE	3/2	5	325	0.37	+27
DOSP	7.5	Chol	1/1	10	Nm	Nm	Nm
DOSP	7.5	DOPI	1/1	2.5	75	0.45	+55
DOSP	7.5	DOPI	1/1	5	77	0.55	+55
DOSP	7.5	DOPI	1/1	10	121	0.33	+53
DOSP	7.5	DOPI	1/1	15	81	0.47	+54
DOSP	7.5	DOPE	1/1	2.5	426	0.43	Nd
DOSP	7.5	DOPE	1/1	5	388	0.47	Nd
DOSP	7.5	DOPE	1/1	10	344	0.51	Nd
DOSP	7.5	DOPE	1/1	15	286	0.42	Nd
DOSP	7.5	Tetraether	5/1	2.5	95	0.27	+61
DOSP	7.5	Tetraether	5/1	5	95	0.27	+63
DOSP	7.5	Tetraether	5/1	10	93	0.27	+50
DOSP	7.5	Tetraether	5/1	15	92	0.36	+45
DOSP	7.5	Tetraether	2.5/1	2.5	207	0.27	+76
DOSP	7.5	Tetraether	2.5/1	5	202	0.27	+68
DOSP	7.5	Tetraether	2.5/1	10	203	0.25	+72
DOSP	7.5	Tetraether	2.5/1	15	203	0.23	+62
CholP	7.5	DOPI	1/1	2.5	147	0.15	Nd
CholP	7.5	DOPI	1/1	5	151	0.15	Nd
CholP	7.5	DOPI	1/1	10	165	0.17	Nd
CholP	7.5	DOPI	1/1	15	173	0.20	Nd
CholP	7.5	DOPE	1/1	2.5	141	0.29	+62
CholP	7.5	DOPE	1/1	5	144	0.27	+58
CholP	7.5	DOPE	1/1	10	143	0.29	+53
CholP	7.5	DOPE	1/1	15	146	0.28	+52
CholP	7.5	DOPE	1/2	2.5	248	0.20	Nd
CholP	7.5	DOPE	1/2	5	248	0.14	Nd
CholP	7.5	DOPE	1/2	10	257	0.12	Nd
CholP	7.5	DOPE	1/2	15	232	0.16	Nd
CholP	7.5	Tetraether	2.5/1	2.5	229	0.52	+73
CholP	7.5	Tetraether	2.5/1	5	241	0.52	+66
CholP	7.5	Tetraether	2.5/1	10	214	0.74	+57
CholP	7.5	Tetraether	2.5/1	15	176	0.55	+53
CholP	7.5	Diether	1/1	2.5	445	0.40	Nd
CholP	7.5	Diether	1/1	5	439	0.27	Nd
CholP	7.5	Diether	1/1	10	399	0.48	Nd
CholP	7.5	Diether	1/1	15	377	0.39	Nd
CholP	7.5	Diether	1/2	2.5	363	0.42	Nd
CholP	7.5	Diether	1/2	5	340	0.43	Nd
CholP	7.5	Diether	1/2	10	339	0.39	Nd
CholP	7.5	Diether	1/2	15	333	0.51	Nd

a) in mM; b), in addition to DP5K; c) Cationic lipid/colipid(s) molar ratio; d) in mg/mL; e) in nm; f) Polydispersity index; g) in mV; *, DP5K pre-insertion (all lipidic compounds were mixed together before the film hydration step); Nd, not determined; Na, not applicable; Nm, non-measurable (due to precipitation and/or aggregation).

Table S2. Composition and physicochemical characterizations of additional lipoplexes performed (but not tested in animals).

Lipid+pDNA formulation composition					Physicochemistry		
**CL**	**Colipid(s)[a]**	**MR[b]**	**PR[c]**	**CR[d]**	**Size[e]**	**PdI[f]**	**Zeta[g]**
DOPAs	DOPE	1/1	1	2	245	0.50	+38
DOGB	Tetraether	10/0.5	1	2	190	0.32	+28
DOSP	DOPI	1/1	0.5	2	Nm	Nm	Nm
DOSP	DOPI	1/1	1	2	112	0.43	+5
DOSP	DOPI	1/1	2	2	190	0.38	-1
DOSP	DOPI	1/1	3	2	104	0.31	+4
DOSP	DOPI	1/1	0.5	1	Nm	Nm	Nm
DOSP	DOPI	1/1	1	1	516	0.58	Nd
DOSP	DOPI	1/1	2	1	309	0.52	Nd
DOSP	DOPI	1/1	3	1	258	0.48	Nd
DOSP	DOPE	1/1	0.5	2	Nm	Nm	Nm
DOSP	DOPE	1/1	1	2	Nm	Nm	Nm
DOSP	DOPE	1/1	2	2	222	0.44	Nd
DOSP	DOPE	1/1	3	2	204	0.46	Nd
DOSP	Tetraether	10/1	1	2	Nm	Nm	Nm
DOSP	Tetraether	5/1	0.5	2	Nm	Nm	Nm
DOSP	Tetraether	5/1	1	2	Nm	Nm	Nm
DOSP	Tetraether	5/1	2	2	224	0.63	+3
DOSP	Tetraether	5/1	3	2	228	0.50	-1
DOSP	Tetraether	2.5/1	0.5	2	Nm	Nm	Nm
DOSP	Tetraether	2.5/1	1	2	322	0.36	-2
DOSP	Tetraether	2.5/1	2	2	463	0.45	-9
DOSP	Tetraether	2.5/1	3	2	270	0.41	-2
CholP	DOPI	1/1	0.5	2	Nm	Nm	Nm
CholP	DOPI	1/1	1	2	Nm	Nm	Nm
CholP	DOPI	1/1	2	2	243	0.35	Nd
CholP	DOPI	1/1	3	2	170	0.23	Nd
CholP	DOPE	1/1	0.5	2	Nm	Nm	Nm
CholP	DOPE	1/1	1	2	Nm	Nm	Nm
CholP	DOPE	1/1	2	2	525	0.73	-4
CholP	DOPE	1/1	3	2	278	0.50	-3
CholP	DOPE	1/2	0.5	2	Nm	Nm	Nm
CholP	DOPE	1/2	1	2	Nm	Nm	Nm
CholP	DOPE	1/2	2	2	447	0.23	Nd
CholP	DOPE	1/2	3	2	240	0.22	Nd
CholP	Tetraether	10/1	1	2	Nm	Nm	Nm
CholP	Tetraether	5/1	1	2	Nm	Nm	Nm
CholP	Tetraether	2.5/1	0.5	2	Nm	Nm	Nm
CholP	Tetraether	2.5/1	1	2	Nm	Nm	Nm
CholP	Tetraether	2.5/1	2	2	527	1.00	-2
CholP	Tetraether	2.5/1	3	2	275	0.83	-1
CholP	Diether	1/1	2	2	498	0.33	Nd
CholP	Diether	1/1	3	2	377	0.29	Nd
CholP	Diether	1/2	1	2	440	0.25	Nd
CholP	Diether	1/2	2	2	377	0.25	Nd
CholP	Diether	1/2	3	2	362	0.17	Nd

a), in addition to DP5K; b) Cationic lipid/colipid(s) molar ratio; c) DP5K/pDNA mass ratio; d) Cationic lipid/pDNA charge ratio; e) in nm; f) Polydispersity index; g) in mV; Nd, not determined; Na, not applicable; Nm, non-measurable (due to precipitation and/or aggregation).

Table S3. Physicochemical follow-up during long-term storage at 4 °C of different preparations of two lipidic formulations (without DNA).

GL67/DOPE 1/2 DP5K ([GL67]=15 mM, DP5K=3.75 mg/mL)

#	Time[a]	Size		PDI		Zeta	
		**Mean**	**SD**	**Mean**	**SD**	**Mean**	**SD**
#1	Day 0	294	3	0.27	0.02	+55	1
	15 days	298	5	0.24	0.02	+53	1
	1 month	304	7	0.21	0.06	+50	4
	5 months	281	7	0.25	0.03	+49	1
	8 months	275	3	0.27	0.02	+51	1
#2	Day 0	Nd	Nd	Nd	Nd	Nd	Nd
	4 months	277	11	0.40	0.01	+50	1
	6 months	221	4	0.25	0.01	+49	1

CholP/DOPE 1/2 DP5K ([CholP]=7.5 mM, DP5K=10 mg/mL)

#	Time[a]	Size		PDI		Zeta	
		**Mean**	**SD**	**Mean**	**SD**	**Mean**	**SD**
#1	Day 0	64	1	0.18	0.01	Nd	Nd
	6 months	46	1	0.24	0.01	+39	1
	10 months	44	1	0.23	0.01	+39	2
	15 months	89	2	0.21	0.01	+40	1
	18 months	89	1	0.21	0.01	+49	2
#2	Day 0	145	1	0.35	0.05	Nd	Nd
	2 months	154	2	0.42	0.01	+41	1
	5 months	147	2	0.36	0.04	+42	1

a) Elapsed time after preparation of liposomes; Nd, not determined.

4. Supplementary discussion

The high quantities of materials needed for conducting aerosol experiment in mice is a hurdle to the development of new formulations. Beside production of high amounts of pDNA, chemical syntheses of all the cationic lipids and colipids included in this study (Figures 1 and 2) were optimized, using protocols either previously reported [2,9–19] or modified for this study (see Supplementary results, section 2.2). This facilitated the production of all the compounds needed in gram-scale quantities with high purity levels.

The strategy to conceive formulations for testing *via* aerosol in animals was basically threefold. First, we chose to evaluate formulations involving lipidic compounds encompassing a wide chemical diversity (Figures 1 and 2, and Table 1). Second, the various lipidic formulations performed were selected on the basis of physicochemical criteria, especially homogeneity and colloidal stability, for before as well as after mixing with pDNA. Third, for the vectors yielding interesting results in preliminary aerosol experiments, variations of their formulation were made by modifying parameters such as the type of colipid used, the cationic lipid/colipid molar ratio, the DP5K/pDNA mass ratio, and the cationic lipid/pDNA charge ratio. Hence, starting with quite empirical investigations, we moved towards a more rational strategy resulting in a significant improvement between initially inefficient formulations to some that were as efficient as, or more efficient than, GL67A in terms of luciferase expression intensity. As for physicochemical examinations prior to testing in animals, we hypothesized that homogeneous, well-characterized formulations (i.e. featuring small size, slightly positive zeta potential, low polydispersity index (PdI), and colloidal stability) should be preferred as they would be both easier to handle and more compliant with pharmaceutical-grade requirements. As regards liposomes, we arbitrarily considered formulations yielding $PdI<0.5$ and $size<300$ nm (Tables 2 and S1). They were supplemented with DP5K, for both improving lipidic mixing and avoiding aggregation/precipitation when blended with a high dose of pDNA. Provided this steric stabilizer was used at a sufficient amount (i.e. for $PR\geq1$), most lipidic formulations could be mixed with pDNA to form homogenous solutions (Tables 3 and S2). However, no relationship was found between homogeneity or colloidal stability of a given lipid+pDNA formulation and its ability to perform gene transfer to the lungs (p-value>0.05). When considering different lipidic formulations, heterogeneous mixtures could indeed yield high gene transfection of the lungs, whereas others featuring both homogeneity and stability demonstrated poor or no effects. Similarly, as for the zeta potential, no relationship was found with transfection efficiency *via* aerosol in lungs (p-value>0.05). Furthermore, all formulations demonstrated a good ability to condense and protect pDNA during nebulization (Figure S2), effectively ruling out pDNA degradation/inactivation as a possible reason for the inefficiency observed with some formulations. Altogether, these results suggest that for a given formulation, the physicochemical profile is not predictive of the aerosol lung gene transfer efficiency; hence, it alone cannot be considered for selecting formulations to be tested in animals. Furthermore, mixtures containing single populations of lipid/pDNA complexes may be unsuitable for reaching the successive stages of the lung airways [20]. Furthermore, it should be stressed that the size of lipid/pDNA complexes must be distinguished from the size of aerosol droplets, the latter depending mainly on the nebulizer used (according to the manufacturer, the PARI LC PLUS generates 5 µm droplets). Finally, these findings suggest that aerosol lung gene delivery was primarily related to intrinsic properties of the lipidic compounds considered.

5. References

1. Cozens, A.L.; Yezzi, M.J.; Kunzelmann, K.; Ohrui, T.; Chin, L.; Eng, K.; Finkbeiner, W.E.; Widdicombe, J.H.; Gruenert, D.C. CFTR Expression and Chloride Secretion in Polarized Immortal Human Bronchial Epithelial Cells. *Am. J. Respir. Cell Mol. Biol.* **1994**, *10*, 38–47.
2. Le Gall, T.; Barbeau, J.; Barrier, S.; Berchel, M.; Lemiègre, L.; Jeftić, J.; Meriadec, C.; Artzner, F.; Gill, D.R.; Hyde, S.C.; et al. Effects of a Novel Archaeal Tetraether-Based Colipid on the in Vivo Gene Transfer Activity of Two Cationic Amphiphiles. *Mol. Pharm.* **2014**, *11*, 2973–2988, doi:10.1021/mp4006276.
3. Lindberg, M.F.; Le Gall, T.; Carmoy, N.; Berchel, M.; Hyde, S.C.; Gill, D.R.; Jaffrès, P.-A.; Lehn, P.; Montier, T. Efficient in Vivo Transfection and Safety Profile of a CpG-Free and Codon Optimized Luciferase Plasmid Using a Cationic Lipophosphoramidate in a Multiple Intravenous Administration Procedure. *Biomaterials* **2015**, *59*, 1–11, doi:10.1016/j.biomaterials.2015.04.024.
4. Nair, D.; Memmi, G.; Hernandez, D.; Bard, J.; Beaume, M.; Gill, S.; Francois, P.; Cheung, A.L. Whole-Genome Sequencing of Staphylococcus Aureus Strain RN4220, a Key Laboratory Strain Used in Virulence Research, Identifies Mutations That Affect Not Only Virulence Factors but Also the Fitness of the Strain. *J. Bacteriol.* **2011**, *193*, 2332–2335, doi:10.1128/JB.00027-11.
5. Baba, T.; Bae, T.; Schneewind, O.; Takeuchi, F.; Hiramatsu, K. Genome Sequence of Staphylococcus Aureus Strain Newman and Comparative Analysis of Staphylococcal Genomes: Polymorphism and Evolution of Two Major Pathogenicity Islands. *J. Bacteriol.* **2008**, *190*, 300–310, doi:10.1128/JB.01000-07.
6. Le Gall, T.; Berchel, M.; Le Hir, S.; Fraix, A.; Salaün, J.Y.; Férec, C.; Lehn, P.; Jaffres, P.-A.; Montier, T. Arsonium-Containing Lipophosphoramides, Poly-Functional Nano-Carriers for Simultaneous Antibacterial Action and Eukaryotic Cell Transfection. *Adv. Healthc. Mater.* **2013**, doi:10.1002/adhm.201200478.
7. Debnath, S.; Bergamini, J.-F.; Artzner, F.; Mériadec, C.; Camerel, F.; Fourmigué, M. Near-Infrared Chiro-Optical Effects in Metallogels. *Chem. Commun. Camb. Engl.* **2012**, *48*, 2283–2285, doi:10.1039/c2cc16549j.
8. Hyde, S.C.; Pringle, I.A.; Abdullah, S.; Lawton, A.E.; Davies, L.A.; Varathalingam, A.; Nunez-Alonso, G.; Green, A.M.; Bazzani, R.P.; Sumner-Jones, S.G.; et al. CpG-Free Plasmids Confer Reduced Inflammation and Sustained Pulmonary Gene Expression. *Nat. Biotechnol.* **2008**, *26*, 549–551.
9. Mével, M.; Sainlos, M.; Chatin, B.; Oudrhiri, N.; Hauchecorne, M.; Lambert, O.; Vigneron, J.-P.; Lehn, P.; Pitard, B.; Lehn, J.-M. Paromomycin and Neomycin B Derived Cationic Lipids: Synthesis and Transfection Studies. *J. Control. Release Off. J. Control. Release Soc.* **2012**, *158*, 461–469, doi:10.1016/j.jconrel.2011.12.019.
10. Mével, M.; Neveu, C.; Goncalves, C.; Yaouanc, J.J.; Pichon, C.; Jaffrès, P.A.; Midoux, P. Novel Neutral Imidazole-Lipophosphoramides for Transfection Assays. *Chem. Commun. Camb. Engl.* **2008**, 3124–3126.
11. Picquet, E.; Le Ny, K.; Delépine, P.; Montier, T.; Yaouanc, J.J.; Cartier, D.; des Abbayes, H.; Férec, C.; Clément, J.C. Cationic Lipophosphoramidates and Lipophosphoguanidines Are Very Efficient for in Vivo DNA Delivery. *Bioconjug. Chem.* **2005**, *16*, 1051–1053.
12. Gonçalves, C.; Berchel, M.; Gosselin, M.-P.; Malard, V.; Cheradame, H.; Jaffrès, P.-A.; Guégan, P.; Pichon, C.; Midoux, P. Lipopolyplexes Comprising Imidazole/Imidazolium Lipophosphoramidate, Histidinylated Polyethyleneimine and SiRNA as Efficient Formulation for SiRNA Transfection. *Int. J. Pharm.* **2014**, *460*, 264–272, doi:10.1016/j.ijpharm.2013.11.005.
13. Desigaux, L.; Sainlos, M.; Lambert, O.; Chevre, R.; Letrou-Bonneval, E.; Vigneron, J.P.; Lehn, P.; Lehn, J.M.; Pitard, B. Self-Assembled Lamellar Complexes of SiRNA with Lipidic Aminoglycoside Derivatives Promote Efficient SiRNA Delivery and Interference. *Proc Natl Acad Sci U A* **2007**, *104*, 16534–16539.
14. Blanchard, E.L.; Loomis, K.H.; Bhosle, S.M.; Vanover, D.; Baumhof, P.; Pitard, B.; Zurla, C.; Santangelo, P.J. Proximity Ligation Assays for In Situ Detection of Innate Immune Activation: Focus on In Vitro-Transcribed MRNA. *Mol. Ther. Nucleic Acids* **2019**, *14*, 52–66, doi:10.1016/j.omtn.2018.11.002.
15. Bhosle, S.M.; Loomis, K.H.; Kirschman, J.L.; Blanchard, E.L.; Vanover, D.A.; Zurla, C.; Habrant, D.; Edwards, D.; Baumhof, P.; Pitard, B.; et al. Unifying in Vitro and in Vivo IVT MRNA Expression Discrepancies in Skeletal Muscle via Mechanotransduction. *Biomaterials* **2018**, *159*, 189–203, doi:10.1016/j.biomaterials.2018.01.010.
16. Lindsay, K.E.; Bhosle, S.M.; Zurla, C.; Beyersdorf, J.; Rogers, K.A.; Vanover, D.; Xiao, P.; Araínga, M.; Shirreff, L.M.; Pitard, B.; et al. Visualization of Early Events in MRNA Vaccine Delivery in Non-Human Primates via PET-CT and near-Infrared Imaging. *Nat. Biomed. Eng.* **2019**, *3*, 371–380, doi:10.1038/s41551-019-0378-3.
17. Habrant, D.; Peuziat, P.; Colombani, T.; Dallet, L.; Gehin, J.; Goudeau, E.; Evrard, B.; Lambert, O.; Haudebourg, T.; Pitard, B. Design of Ionizable Lipids To Overcome the Limiting Step of Endosomal Escape: Application in the Intracellular Delivery of MRNA, DNA, and SiRNA. *J. Med. Chem.* **2016**, *59*, 3046–3062, doi:10.1021/acs.jmedchem.5b01679.
18. Réthoré, G.; Montier, T.; Le Gall, T.; Delépine, P.; Cammas-Marion, S.; Lemiègre, L.; Lehn, P.; Benvegnu, T. Archaeosomes Based on Synthetic Tetraether-like Lipids as Novel Versatile Gene Delivery Systems. *Chem. Commun. Camb. Engl.* **2007**, 2054–2056.
19. Demazeau, M.; Quesnot, N.; Ripoche, N.; Rauch, C.; Jeftić, J.; Morel, F.; Gauffre, F.; Benvegnu, T.; Loyer, P. Efficient Transfection of Xenobiotic Responsive Element-Biosensor Plasmid Using Diether Lipid and Phosphatidylcholine Liposomes in Differentiated HepaRG Cells. *Int. J. Pharm.* **2017**, *524*, 268–278, doi:10.1016/j.ijpharm.2017.03.080.
20. Resnier, P.; Mottais, A.; Sibiril, Y.; Le Gall, T.; Montier, T. Challenges and Successes Using Nanomedicines for Aerosol Delivery to the Airways Available online: https://pubmed-ncbi-nlm-nih-gov.proxy.insermbiblio.inist.fr/26725879/?from_term=resnier+p&from_pos=2 (accessed on 2 June 2020).

6. List of abbreviations

BLI	bioluminescence imaging
CF	cystic fibrosis
Chol	cholesterol
CholAs	cholesterol arsonium
CholIm	cholesterol imidazolium
CholK	cholesterol kanamycin A
CholKB	cholesterol kanamycin B
CholP	cholesterol paromomycin
CholRi	cholesterol ribostamycin
CholT	cholesterol tobramycin
CL	cationic lipid
CR	charge ratio
DOGB	dioleyl glycine betaine
DOPAs	dioleyl phosphoramide arsonium
DOPE	dioleyl phosphatidylethanolamine
DOPI	dioleyl phosphoramide imidazole
DOPIm	dioleyl phosphoramide imidazolium
DOSP	dioleyl-succinyl paromomycin
DP5K	1,2-dimyristoyl-sn-glycero-3-phosphoethanolamine conjugated polyethylene glycol 5000
GL67	Genzyme lipid 67
PdI	polydispersity index
MLE	maximal luciferase expression
RT	room temperature
